# A bidirectional Mendelian randomization study of sarcopenia-related traits and inflammatory bowel diseases

**DOI:** 10.3389/fimmu.2023.1240811

**Published:** 2023-11-08

**Authors:** Xin Jiao, Wen-yu Wu, Shao-feng Zhan, Jian-bo Liu, Xian-jin Zhang

**Affiliations:** ^1^ The First Clinical Medical College of Guangzhou University of Chinese Medicine, Guangzhou, China; ^2^ The First Affiliated Hospital of Guangzhou University of Chinese Medicine, Guangzhou, China

**Keywords:** sarcopenia, inflammatory bowel disease, Crohn’s disease, ulcerative colitis, Mendelian randomization, causal relationship

## Abstract

**Background:**

There is increasing evidence pointing to a close relationship between sarcopenia and inflammatory bowel disease. However, it remains unclear whether or in which direction causal relationships exist, because these associations could be confounded.

**Methods:**

We conducted a two-sample bidirectional mendelian randomization analysis using data from European genome-wide association studies of the appendicular lean mass(n = 450,243), walking pace(n = 459,915), grip strength (left hand, n = 461,026; right hand, n = 461,089), inflammatory bowel disease (25,042 patients and 34,915 controls), ulcerative colitis (12,366 patients and 33,609 controls), and Crohn’s disease (12,194 patients and 28,072 controls) to investigate the causal relationship between sarcopenia-related traits and inflammatory bowel disease and its subtypes on each other. The inverse-variance weighted method was used as the primary analysis method to assess the causality, and a comprehensive sensitivity test was conducted.

**Results:**

Genetically predicted appendicular lean mass was significantly associated with inflammatory bowel disease (OR = 0.916, 95%CI: 0.853–0.984, *P* = 0.017), ulcerative colitis (OR =0.888, 95%CI: 0.813–0.971, *P* = 0.009), and Crohn’s disease (OR = 0.905, 95%CI: 0.820–0.999, *P* = 0.049). Similar results also revealed that the usual walking pace was causally associated with Crohn’s disease (OR = 0.467, 95%CI: 0.239–0.914, *P* = 0.026). Reverse mendelian randomization analysis results found that genetic susceptibility to inflammatory bowel disease, and Crohn’s disease were associated with lower appendicular lean mass. A series of sensitivity analyses ensured the reliability of the present research results.

**Conclusion:**

The mendelian randomization study supports a bidirectional causality between inflammatory bowel disease, Crohn’s disease and appendicular lean mass, but no such bidirectional causal relationship was found in ulcerative colitis. In addition, genetically predicted usual walking pace may reduce the risk of Crohn’s disease. These findings have clinical implications for sarcopenia and inflammatory bowel disease management.

## 1 Introduction

Sarcopenia is a gradual and systemic skeletal muscle disease characterized by a loss of skeletal muscle mass or function that happens regularly with aging and is associated with an increased risk of poor outcomes such as falls, disability, impaired function, frailty, hospitalization, and mortality (1–4). Sarcopenia was classified as a disease by the World Health Organization in 2016 and given an International Classification of Diseases-10 code (5). Sarcopenia can be primary (age-related) or accompany a variety of chronic illnesses in middle age appendiceal skeletal muscle mass, such as inflammation, malnutrition, neoplastic disease, or organ failure (1, 6, 7). According to estimates, sarcopenia affects 10% to 16% of aged people worldwide (8). Along with older persons, those who are underweight, women, and those who have other chronic ailments are more susceptible to developing sarcopenia and its detrimental effects on their health (9). Sarcopenia incidence is rising quickly as the world’s population ages (10, 11), and by 2050, 500 million people are expected to have the condition (12).

Inflammatory bowel disease (IBD) is an idiopathic inflammatory illness of the gastrointestinal tract that comprises Crohn’s disease (CD) and ulcerative colitis (UC) (13). Over the last decade, the prevalence of IBD has increased worldwide (14). Muscle loss is a typical pathophysiological aspect of many chronic gastrointestinal illnesses, including IBD (15). When compared to healthy individuals, patients with IBD have a lower lean body mass composition and reduced muscle mass by up to 60% (14, 16). Even at a young age, patients with IBD might develop sarcopenia (17). Sarcopenia may exist even in IBD patients in remission (18). When IBD patients are divided into those who are UC or CD, those who are CD have a higher frequency of sarcopenia than those who are UC (52% vs. 37%, respectively) (17). Sarcopenia affects up to 50% of IBD patients and is related to adverse clinical outcomes (19), such as longer hospitalization, intestinal resection, and an increased risk of post-operative complications (15, 20, 21).

Several previous studies have shown that patients with sarcopenia have a higher risk of developing IBD and vice versa (8, 15, 17, 18, 22). However, Pedersen et al. found no difference in the incidence of sarcopenia between people with IBD and those without IBD (23). The relationship between sarcopenia and IBD is unclear and controversial, and their genetically predicted causal effect remains unexplored. Here, we aim to evaluate the causality between Sarcopenia-related traits and IBD and its subtypes (UC and CD) using a two-sample Mendelian randomization (MR) design. MR is a genetic epidemiological research method that greatly improves the ability to investigate the causal relationship between traits and diseases because it addresses some of the common drawbacks of traditional epidemiological studies (such as reverse causation) and ensures that results are unaffected by confounding factors (24, 25). This study was conducted in both directions (i.e. bidirectional MR), as this is also an advantageous strategy to orient the direction of causal effects between two variables (26).

## 2 Materials and methods

Using a two-sample MR analytic approach, which is an extension of MR where the effects of genetic instruments on exposure and outcome are extracted from two different datasets, we evaluated the causality between sarcopenia-related characteristics and IBD and its subtypes (CD and UC) (27).The three core MR assumptions for assuming causal relationship are as follows (1): the genetic variants have a strong connection to the exposure (2); there don’t exist unmeasured confounders of the associations between outcome and genetic variants; and (3) the genetic variants effect the outcome just via the exposure (28).

**Figure 1:**
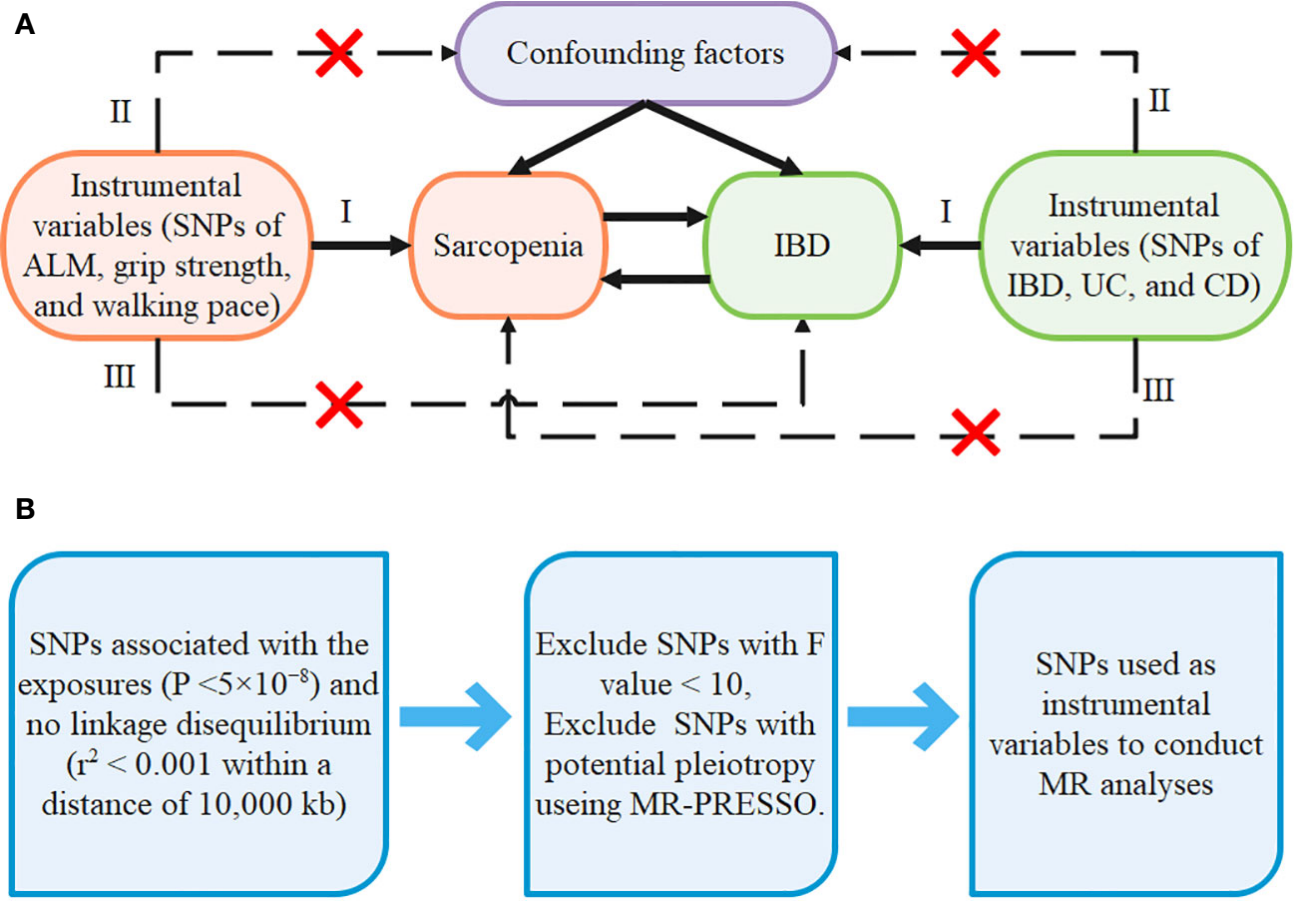
The design of our MR framework **(A)** Flowchart of the bidirectional Mendelian randomization study. **(B)** Flow diagram of SNPs screening.

Due to the fact that the current study is a secondary analysis of data that is available to the public, no separate ethical approval was necessary. The GWAS summary statistics are accessible at https://gwas.mrcieu.ac.uk/. The details of GWAS used to get the summary statistics for each trait are listed in **Supplementary Table 1**. The flowchart of the bidirectional MR design is shown in **Figure 1A**.

### 2.1 GWAS summary statistics of sarcopenia-related traits

We collected three functional parameters as sarcopenia-related traits, including appendicular lean mass (ALM), usual walking pace, and handgrip strength (left & right), since they are reliable predictors of sarcopenia (7).

As the most regularly used approximate index of muscle mass in sarcopenia research, ALM is regarded a reliable measure of muscle mass and is frequently used in the Asian Working Group for Sarcopenia (AWGS) and European Working Group on Sarcopenia in Older People (EWGSOP) diagnostic criterion for sarcopenia (29–31). The GWAS data of ALM was acquired from a meta-analysis of 450,243 UK Biobank participants (32), and ALM was measured using bioelectrical impedance analysis (33).

Since low physical performance is one of the characteristics of sarcopenia, gait speed measurement is also an essential diagnostic criteria for sarcopenia (34). In clinical practice, the walking pace is frequently employed as a quick, secure, and highly reliable test for sarcopenia (7). The summary statistics used as genetic predictors of walking pace were obtained from the UK Biobank, which comprises 459,915 individuals (35).

Grip strength is a widely used proxy of muscle fitness that can serve as a trustworthy alternative for overall muscle strength (7, 36, 37). Because the correlation between absolute handgrip strength and muscle strength may be higher than that of relative grip strength (38), we used absolute handgrip strength as a proxy for muscle strength. The UK Biobank provided the GWAS summary statistics for handgrip strength (left hand, n = 461,026; right hand, n = 461,089) (35). Handgrip strength was measured by adjusting hand dimensions with a calibrated hydraulic hand dynamometer (39).

### 2.2 GWAS summary statistics of IBD and its subtypes

The IEU Open GWAS database was used to acquire summary-level data for IBD and its subtypes (CD and UC). In the IBD data, there were 25,042 patients and 34,915 controls, and in the UC and CD data, there were 12,366 and 12,194 patients, respectively (40). The IBD data have included UC and CD subsets, the detailed table for the summary of UC, CD and IBD original data samples can be seen in the **Supplementary Table 2**, which was downloaded from the original literature (40). These diagnoses are based on radiographic, endoscopic, and histological criteria that are widely accepted (41). All study participants were of European ancestry to avoid pleiotropic deviation of cross-lineage cases (42).

### 2.3 Selection of instrumental variables

For each MR analysis, we selected all single nucleotide polymorphisms (SNPs) associated with the exposure as instrumental variables. First, we selected SNPs at a genome-wide significance threshold (p<5×10^-8^) and there is no linkage disequilibrium at the selected SNPs (r^2^< 0.001 within a distance of 10,000 kb) (42, 43). Second, SNPs related to confounders and outcomes were excluded. We calculated the F-statistic to ensure the intensity of exposure, where a value greater than 10 is considered sufficiently robust to offset weak instrument bias (44), therefore the SNPs with F-statistic less than 10 were removed. The formula for calculating the SNPs’ R^2^ and F-statistics are as follows (45). Third, outliers were detected using MR-PRESSO models, and if any were found, they were deleted and re-analyzed using the remaining SNPs. Details of the SNPs used as instrumental variables in each MR analysis are available in **Supplementary Tables 3**, **4**. The flow diagram of SNPs screening is shown in **Figure 1B**.


$$R^{2}\;=\;2\times EAF\times \left(1-EAF\right)\times \beta^{2},\;F\;statistic=R^{2}\times \left(N-2\right)/\left(1-R^{2}\right)$$

### 2.4 Statistical analyses

We selected inverse-variance weighted (IVW) MR with multiplicative random-effects as the main analysis method because it provides the most efficient combination of variant specificity ratio estimates and takes into account the heterogeneity of causal estimates obtained from individual variants (42, 45, 46). The results of IVW were visualized using forest plots. We also used four other additional MR analytical methods, including weighted median, MR-Egger, weighted mode, and simple mode, which allowed horizontal pleiotropy with a lower statistical capability than IVW.

In addition, a series of additional sensitivity analysis methods were performed to ensure the robustness of the results. We assessed the presence of heterogeneity amongst the variant-specific causal estimates using Cochran’s Q statistic and generated funnel plots (47, 48). p<0.05 values indicate significant heterogeneity and the funnel plot is expected to be symmetrical (49). MR-egger regression was used to test the pleiotropy and leave-one-out analysis was used to see if there was any bias due to individual SNP influencing the findings independently (50). Additionally, the MR-PRESSO method was used to detect outliers and identify horizontal pleiotropy, and if abnormal SNPs were detected, they were removed to eliminate pleiotropy effects (51).

All statistical analyses and results visualization were performed in R software (Version 4.2.3, https://www.r-project.org/) with the help of “two-sample MR” (v.0.5.1), “MR-PRESSO” (v.1.0) and “Forest plot” packages.

## 3 Results

### 3.1 Causal relationship of sarcopenia-related traits on IBD and its subtypes

**Figure 2:**
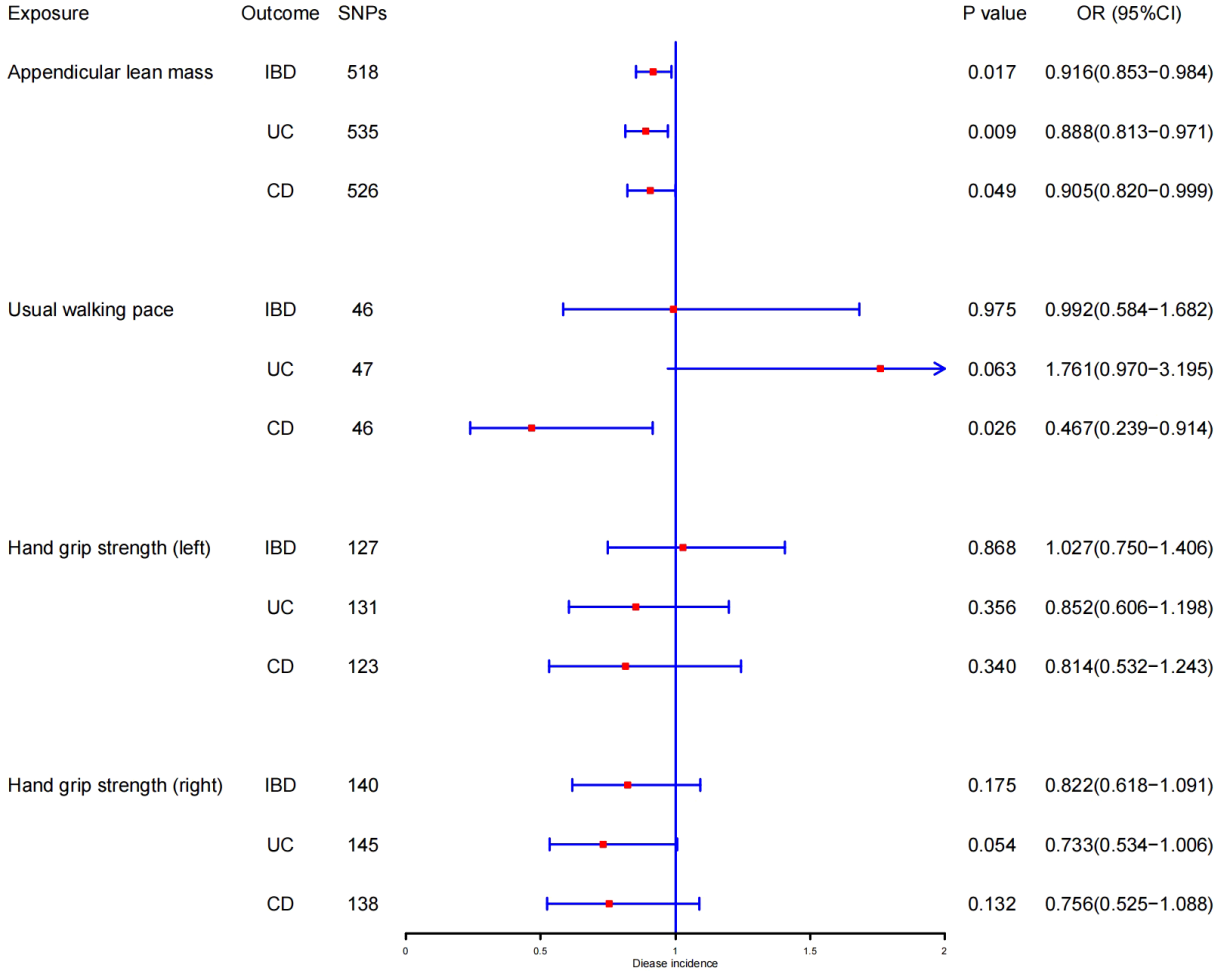
MR estimates using the IVW method of ALM, hand grip strength (right & left), and usual walking pace on IBD, UC, and CD. SNPs, single-nucleotide polymorphisms; OR, odds ratio; CI, confidence interval.

**Figure 3:**
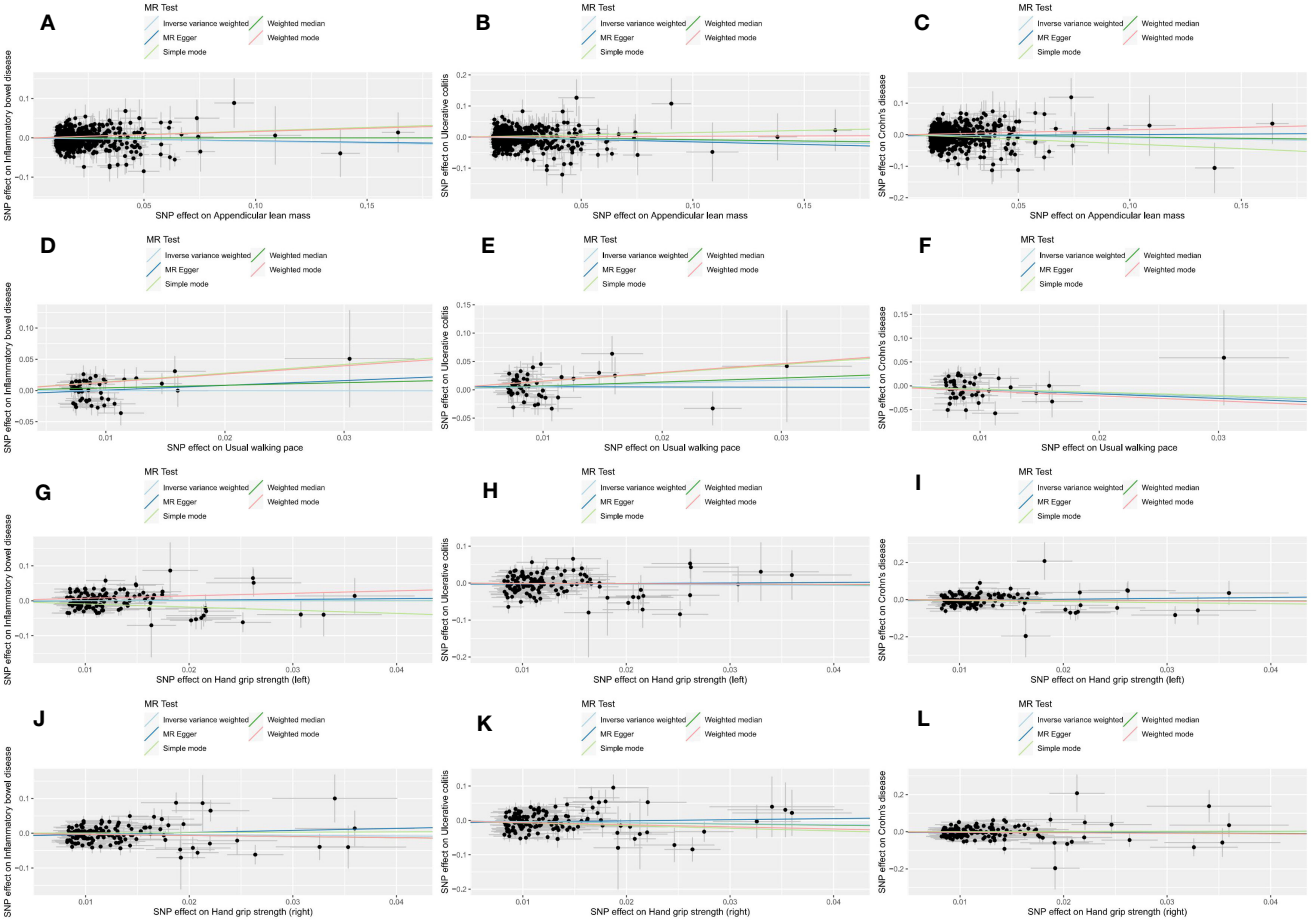
Scatter plots of forward MR analysis. The slope of each line represents the expected MR impact in various models. **(A)** ALM on IBD; **(B)** ALM on UC; **(C)** ALM on CD; **(D)** usual walking pace on IBD; **(E)** usual walking pace on UC; **(F)** usual walking pace on CD;**(G)** hand grip strength (left) on IBD; **(H)** hand grip strength (left) on UC; **(I)** hand grip strength (left) on CD; **(J)** hand grip strength (right) on IBD; **(K)** hand grip strength (right) on UC; **(L)** hand grip strength (right) on CD.

In the forward MR analysis, the results of estimating the causal effects of sarcopenia-related traits on IBD and its subtypes using the IVW method were shown in **Figure 2**. Scatter plots were provided in **Figure 3**. Genetically predicted ALM was significantly associated with IBD (OR = 0.916, 95%CI: 0.853–0.984, P = 0.017), UC (OR =0.888, 95%CI: 0.813–0.971, P = 0.009), and CD (OR = 0.905, 95%CI: 0.820–0.999, P = 0.049). We also found that the usual walking pace was causally associated with CD (OR = 0.467, 95%CI: 0.239–0.914, P = 0.026). There was no evidence to support the association between handgrip strength (right & left) with IBD and its subtypes (UC and CD). We discovered evidence of heterogeneity using Cochran’s Q, indicating that the IVW with random effects approach was acceptable (47). The MR-Egger intercept test did not provide any indications of directional pleiotropy (**Table 1**), and the funnel plot did not reveal any signs of symmetry deviation either [**Supplementary File 1(A-L)**]. In leave-one-out sensitivity analyses, the overall IVW estimates did not change on the exclusion of any variant [**Supplementary File2(A-L)**]. Overall, the higher appendicular lean mass is a protective factor for IBD and its subtypes, and a genetically elevated usual walking pace may reduce the risk of CD.

**Table 1:** Sensitivity analysis of the forward MR results.

Exposure	Outcome	Method	Cochran's Q	Q_pval	MR-Egger Intercept	P-intercept
Appendicular lean mass	IBD	MR Egger	751.1	5.50E-11		
		IVW	751.16	6.60E-11	-0.000403208	0.83
	UC	MR Egger	764.09	1.75E-10		
		IVW	764.45	1.99E-10	0.001156166	0.617
	CD	MR Egger	857.06	1.75E-18		
		IVW	859.44	1.40E-18	-0.003119143	0.228
Usual walking pace	IBD	MR Egger	60.03	5.42E-02		
		IVW	60.46	6.15E-02	-0.006980221	0.575
	UC	MR Egger	50.97	2.50E-01		
		IVW	51.25	2.75E-01	0.005667504	0.624
	CD	MR Egger	58.41	7.16E-02		
		IVW	58.42	8.64E-02	0.001565634	0.921
Hand grip strength (left)	IBD	MR Egger	254.34	7.43E-11		
		IVW	254.51	1.02E-10	-0.002162404	0.772
	UC	MR Egger	200.21	5.93E-05		
		IVW	200.52	7.07E-05	-0.003552886	0.656
	CD	MR Egger	255.56	1.19E-11		
		IVW	257	1.19E-11	-0.008161921	0.411
Hand grip strength (right)	IBD	MR Egger	251.99	1.10E-08		
		IVW	255.62	6.39E-09	-0.009339798	0.161
	UC	MR Egger	210.52	2.05E-04		
		IVW	212.19	1.90E-04	-0.007707431	0.289
	CD	MR Egger	241.18	7.20E-08		
		IVW	241.94	8.17E-08	-0.005612354	0.515

### 3.2 Causal relationship of IBD and its subtypes on sarcopenia-related traits

**Figure 4:**
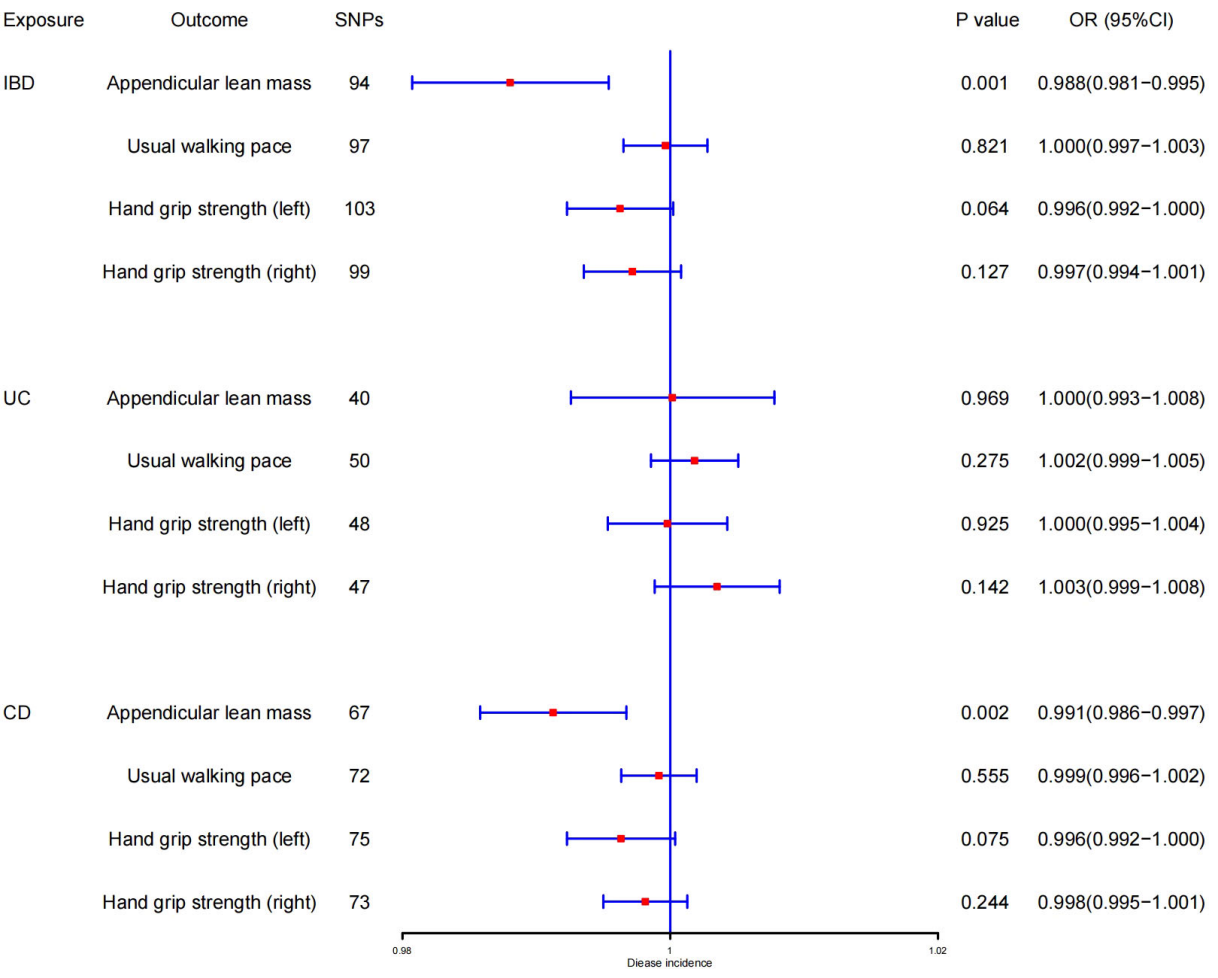
MR estimates using the IVW method of IBD, UC, and CD on ALM, hand grip strength (right & left), and usual walking pace. SNPs, single-nucleotide polymorphisms; OR, odds ratio; CI, confidence interval.

**Figure 5:**
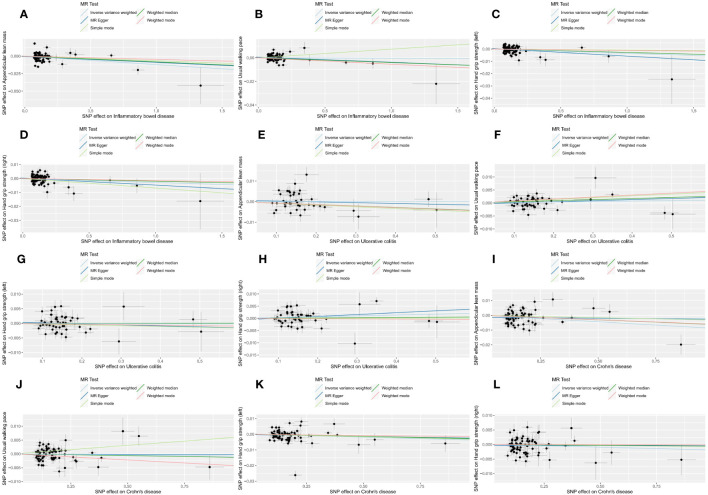
Scatter plots of reverse MR analysis. The slope of each line represents the expected MR impact in various models. **(A)** IBD on ALM; **(B)** IBD on usual walking pace; **(C)** IBD on hand grip strength (left); **(D)** IBD on hand grip strength (left); **(E)** UC on ALM; **(F)** UC on usual walking pace;**(G)** UC on hand grip strength (left); **(H)** UC on hand grip strength (right); **(I)** CD on ALM; **(J)** CD on usual walking pace; **(K)** CD on hand grip strength (left); **(L)** CD on hand grip strength (right).

The outcomes of reverse MR analysis were displayed in **Figure 4**. The scatter plots were provided in **Figure 5**. Genetically predicted IBD and CD were significantly related with lower ALM, (OR = 0.988, 95%CI: 0.981–0.995, *P* = 0.001) and (OR = 0.991, 95%CI:0.986–0.997, *P* = 0.002), respectively. However, there was no correlation between UC and ALM (p > 0.05). The IVW results also indicated that IBD, UC, and CD had no causal effect on the usual walking pace and handgrip strength (left & right). Sensitivity analysis revealed no significant horizontal pleiotropy (**Table 2**). The funnel plot and leave-one-out sensitivity analysis can be found in the **Supplementary Files 1(M-X)**, **2(M-X)**. In other words, UC will not increase the risk of ALM, and there is a positive correlation between genetic vulnerability to IBD, CD, and the risk of ALM.

**Table 2:** Sensitivity analysis of the reverse MR results.

Exposure	Outcome	Method	Cochran's Q	Q_pval	MR-Egger Intercept	P-intercept
IBD	Appendicular lean mass	MR Egger	395.68	2.80E-39		
		IVW	396.66	4.00E-39	-0.000506481	0.634
	Usual walking pace	MR Egger	168.7	4.90E-06		
		IVW	171.25	3.69E-06	0.000540609	0.234
	Hand grip strength (left)	MR Egger	225.09	1.89E-11		
		IVW	225.47	2.57E-11	0.000239434	0.683
	Hand grip strength (right)	MR Egger	175.17	2.01E-06		
		IVW	175.75	2.40E-06	0.000300487	0.574
UC	Appendicular lean mass	MR Egger	112.16	3.02E-09		
		IVW	112.52	4.69E-09	0.000664168	0.73
	Usual walking pace	MR Egger	75.3	7.14E-03		
		IVW	75.78	8.39E-03	-0.000404411	0.585
	Hand grip strength (left)	MR Egger	86.73	2.65E-04		
		IVW	87.08	3.42E-04	0.000487766	0.668
	Hand grip strength (right)	MR Egger	99.36	5.63E-06		
		IVW	100.27	6.59E-06	-0.000675366	0.525
CD	Appendicular lean mass	MR Egger	188.66	5.53E-14		
		IVW	192.78	2.39E-14	-0.00137052	0.237
	Usual walking pace	MR Egger	142.41	7.29E-07		
		IVW	142.49	1.04E-06	-0.000121806	0.846
	Hand grip strength (left)	MR Egger	222.31	5.38E-17		
		IVW	222.58	8.63E-17	-0.000263208	0.765
	Hand grip strength (right)	MR Egger	127.7	4.27E-05		
		IVW	128.22	5.16E-05	-0.000367265	0.59

## 4 Discussion

In conclusion, we investigated the causality between sarcopenia and IBD on each other using large-scale GWAS summary data. MR analyses indicated a potential causal relationship between ALM and IBD and revealed that more ALM is a protective factor for IBD, UC, and CD. Furthermore, we found evidence of a potential causal role for the usual walking pace in CD. In the inverse MR analysis, genetically predicted IBD and CD were significantly associated with a lower ALM. No evidence supported the causal relationship between handgrip strength with IBD and its subtypes (UC and CD).

Previous research has demonstrated that a variety of factors, including chronic inflammation, malnutrition, vitamin deficiency, and an imbalance in the muscle-gut axis, might contribute to sarcopenia (52). IBD patients are at risk for weight loss, malnutrition, and long-term glucocorticoid usage, all of which have an adverse effect on muscular strength and mass. The premature and “accelerated” sarcopenia development has been found in other populations affected by factors such as malnutrition, chronic inflammation, and immobility, which are associated with IBD (36). Another study also found that individuals with IBD had fewer skeletal muscle and bone masses, with more visceral and “creeping” fat (53). Saul et al. discovered that experimental IBD mice exhibited lower skeletal muscle weight and fiber size, as well as lower muscle protein content in the gastrocnemius and quadriceps (54). According to numerous investigations, sarcopenia correlates with the severity of IBD and assists in determining treatment decisions for patients with IBD (55–57). In a recent meta-analysis, 52% of CD patients and 37% of UC patients experienced sarcopenia (17). These findings are significant because sarcopenia is known to have a considerable influence on duration of hospital stay, surgical outcomes, life quality, and mortality (22, 41, 58).

In reality, sarcopenia may be a practical and accessible risk assessment tool for people with IBD, such as the necessity for surgical intervention and post-operative complications (6). Currently, there is no curative treatment for CD, whereas UC can be cured by a pan proctocolectomy (59). It can have even more negative effects when sarcopenia and IBD coexist, previous research revealed that patients with CD who were sarcopenic had a higher risk of adverse events and intestinal resection than those who weren’t sarcopenic (15, 60). In a study of 72 IBD patients, sarcopenia significantly increased the risk of intestinal resection (23). The chance of postoperative complications in IBD was shown to be more than six times higher in people with sarcopenia, according to a meta-analysis (61). As a result, sarcopenia is a crucial therapeutic and prognostic consideration in the management of IBD.

Although the exact cause of sarcopenia in IBD patients is still unknown, there is evidence that it may be brought on by drug interactions, malabsorption, poor protein intake, chronic inflammation, and reduced physical activity (62). Recent research has also demonstrated that the insulin-like growth factor 1, phosphoinositide 3 kinase, protein kinase B, and mammalian target of rapamycin (IGF1/PI3K/Akt/mTOR) axis dysregulation is associated with a decrease in IGF1-R in muscle tissues from IBD patients (63). Sarcopenia is frequently associated with malnutrition as a result of chronic persistent inflammation (64). IBD patients usually have poor nutritional status, and it is known that malnutrition is a significant factor in the loss of muscle mass and the ensuing loss of function (58). Although the direct causality between sarcopenia and inflammation has not been established, there is evidence of an association within the context of IBD (65). A study of 441 adults over the age of 60 found that sarcopenic older people had significantly higher levels of tumor necrosis factor (TNF) – α and circulating interleukin (IL)-6 (66).

Sarcopenia involves muscle loss and dysfunction, causing systolic dysfunction, endocrine and metabolic abnormalities, and affecting metabolic and immune/inflammatory responses throughout the body (67). In our MR Results, genetically predicted ALM was strongly associated with IBD and its subtypes. ALM is primarily affected by skeletal muscle mass, which also has endocrine functions that affect inflammatory response and systemic metabolism (67, 68). Low muscle mass is common in IBD patients (69, 70). Studies have shown that levels of pro-inflammatory factors, including CRP, IL-6, and TNF-α, are inversely correlated with ALM (71, 72). Sarcopenia is associated with factors such as progressive increase in fibrosis, chronic inflammatory state, altered muscle metabolism, and degeneration of the neuromuscular junction (73). From a mechanistic perspective, chronic inflammation is most likely associated with sarcopenia leading to IBD. In addition, fatigue and reduced quality of life associated with muscle loss can also contribute to the progression of IBD (74, 75). Studies have also revealed that individuals with IBD had lower grip strength values, however these studies only analyzed the differences in grip strength values between individuals with IBD and controls, not the prevalence of low grip strength (76, 77). In our study, no relationship between IBD and grip strength was found. This may be because IBD is not linked to low grip strength but rather grip strength reduction. However, since recognition of sarcopenia is relatively recent, the mechanism by which sarcopenia leads to IBD remains to be further investigated.

Despite the high prevalence and clinical significance of sarcopenia in IBD patients, few patients receive routine muscle assessment. Furthermore, due to the screening tools are not accurate, most cases of sarcopenia go undetected (78). Given the aging population, an increase in sarcopenia management healthcare costs is to be expected (79). Currently, physical activity is advised as the primary treatment for sarcopenia, and numerous studies have also demonstrated the effectiveness and safety of physical exercise among patients with IBD (7, 80, 81). Additionally, nutritional intervention is also an essential component in treating IBD patients, which may reduce the inflammatory state and the risk of sarcopenia (82, 83).It has also been shown that infliximab therapy can reverse IBD-associated sarcopenia in individuals with active Crohn’s disease, with 6 months of treatment leading to significant improvements in skeletal muscle volume and maximum isokinetic strength (84). Muscle health should be evaluated regularly in all IBD patients, and effective management of sarcopenia in IBD will assist improve prognosis.

This is the first two-sample MR study to investigate the causal association between sarcopenia-related traits and IBD. The main strength of this study is the MR analysis design, which reduces some of the limitations in observational studies, such as confounding and reverse causality. Additionally, sensitivity studies were carried out to assess the robustness of this study’s findings and to assure the consistency of the causal estimations, while several MR analysis techniques were used to verify the accuracy and validity of the results. Notwithstanding the strengths of our study, there are several limitations. First, we employed ALM rather than appendiceal skeletal muscle mass to measure muscle mass because bias in other non-fat soft tissue components such the lung and kidney could affect the results. Second, we were unable to determine the causal link between genetically predicted sarcopenia and IBD in different genders and age groups due to the paucity of data stratified by sex and age. Given that the onset factors of sarcopenia and inflammatory bowel disease are related to age and gender (85–87). Third, the generalizability of our conclusions to other ethnicities may be limited because the results of our study were based on people of European ancestry. Fourth, even if confounding factors were removed in this study, it is difficult to completely remove mediation and pleiotropy, and a larger scale GWAS are needed to verify the results.

## 5 Conclusion

Our MR Results support a bidirectional causal relationship between IBD, CD, and ALM, but such a bidirectional causal relationship was not found in UC. Furthermore, the genetically predicted usual walking pace was causally associated with CD. Our novel insights may provide a better reference for the identification, evaluation, and management of sarcopenia in IBD patients. In order to enhance clinical prognosis, we emphasized that IBD patients should receive proper nutrition and strengthen exercise to combat sarcopenia.

## Data availability statement

The original contributions presented in the study are included in the article/**Supplementary Material**, further inquiries can be directed to the corresponding author/s.

## Author contributions

X-JZ, J-BL and S-FZ conceived and designed this research. XJ and W-YW performed data analyses, interpreted results, and drafted the manuscript. X-JZ reviewed the final manuscript. All authors contributed to the article and approved the submitted version.

## Supplementary material

Supplementary File 1
**(A–L)** Funnel plots of forward MR analysis. **(M–X)** Funnel plots of reverse MR analysis.

Supplementary File 2
**(A–L)** Leave-one-out sensitivity analysis of forward MR. **(M–X)** Leave-one-out sensitivity of reverse MR analysis. **(A–L)** stand for ALM→IBD, ALM→UC, ALM→CD, Usual walking pace →IBD, Usual walking pace →UC, Usual walking pace →CD, Hand grip strength (left)→IBD, Hand grip strength (left)→UC, Hand grip strength (left)→CD, Hand grip strength (right)→IBD, Hand grip strength (right)→UC, Hand grip strength (right)→CD, respectively. **(M–X)** stand for IBD→ALM, IBD →Usual walking pace, IBD →Hand grip strength (left), IBD →Hand grip strength (left),UC→ALM, UC →Usual walking pace, UC →Hand grip strength (left), UC →Hand grip strength (left), UC→ALM, CD →Usual walking pace, CD →Hand grip strength (left), CD →Hand grip strength (left), respectively.
