# Supplementary figures and images for: A bidirectional Mendelian randomization study of sarcopenia-related traits and inflammatory bowel diseases
Source: Front Immunol. 2023 Nov 8;14:1240811. doi: 10.3389/fimmu.2023.1240811 (PMC10666781; doi:10.3389/fimmu.2023.1240811)

A

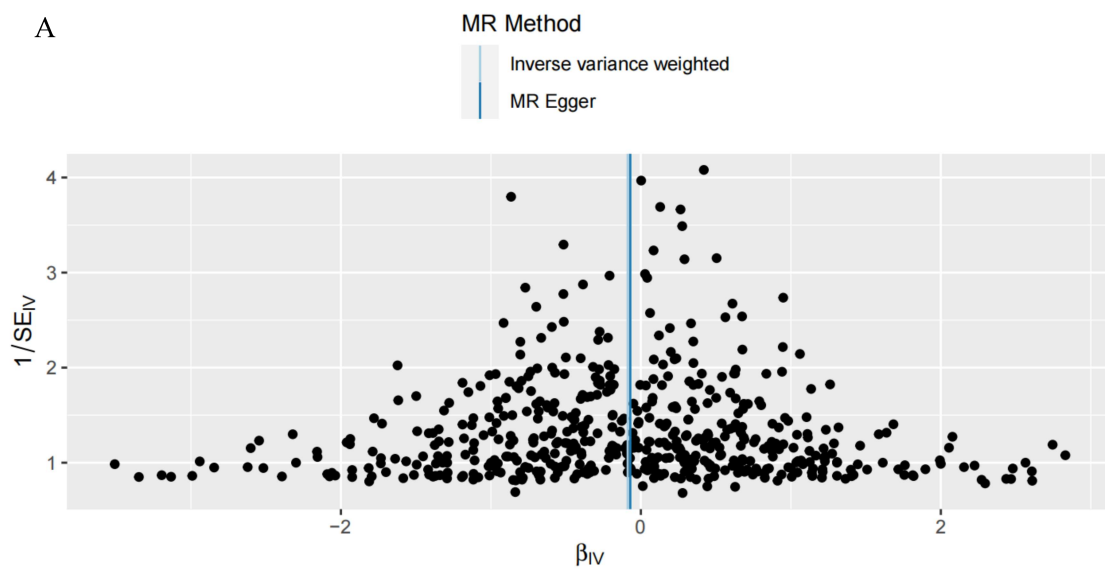

B

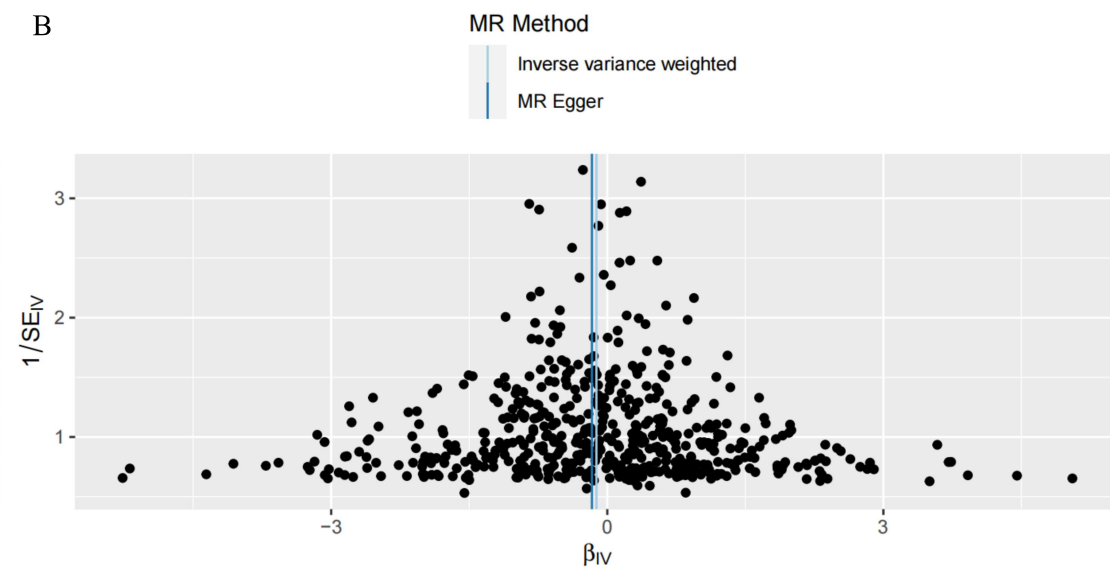

C

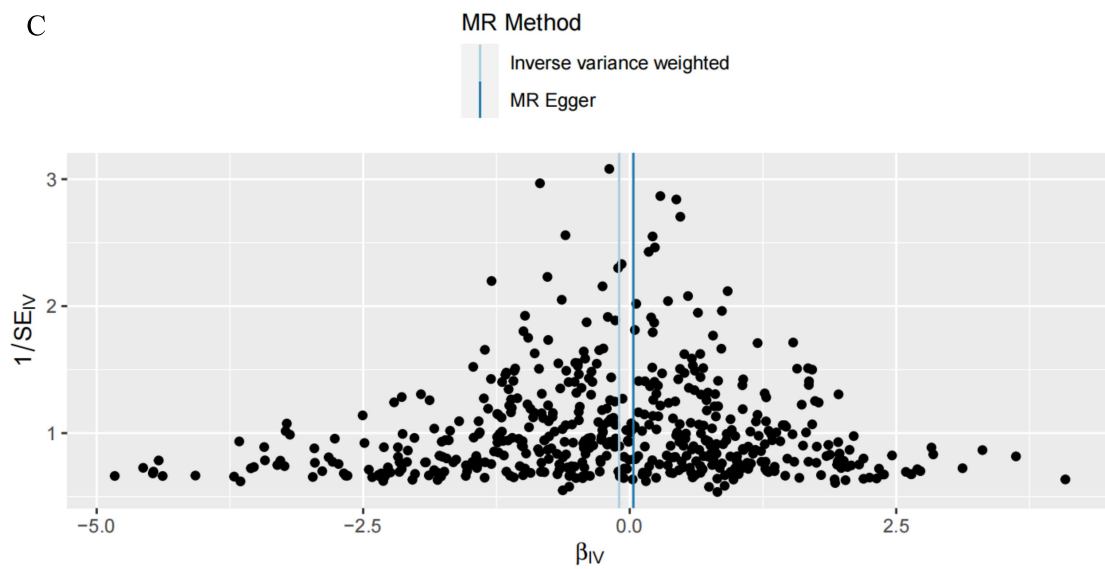

D

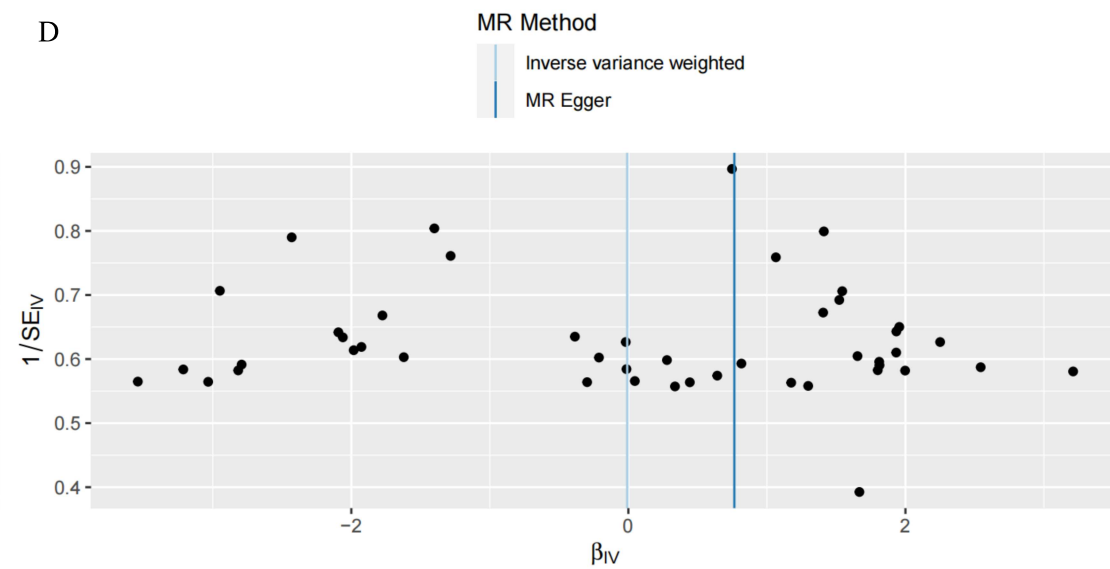

E

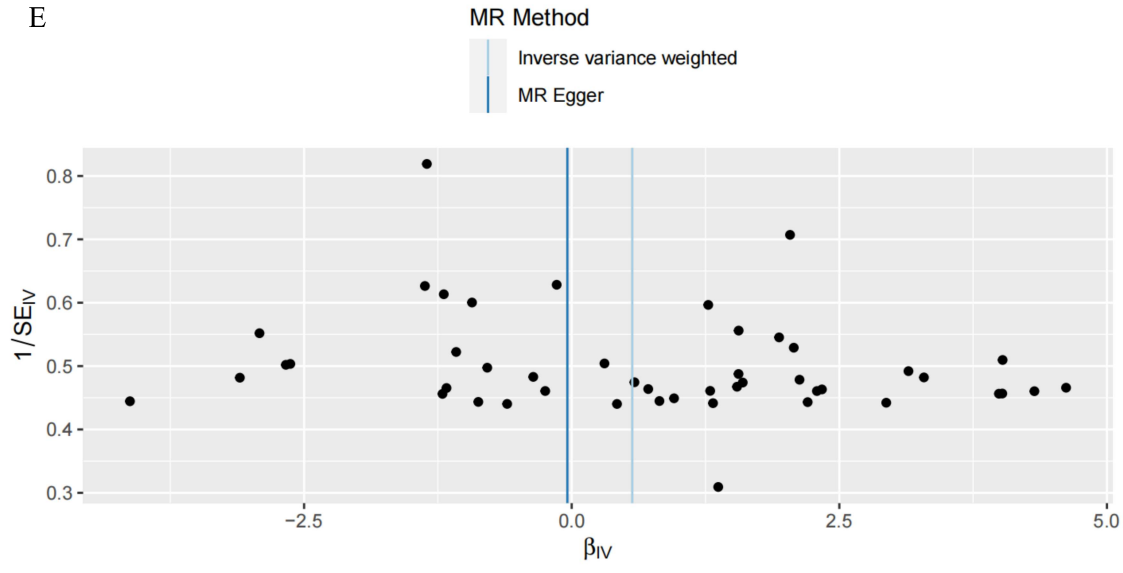

F

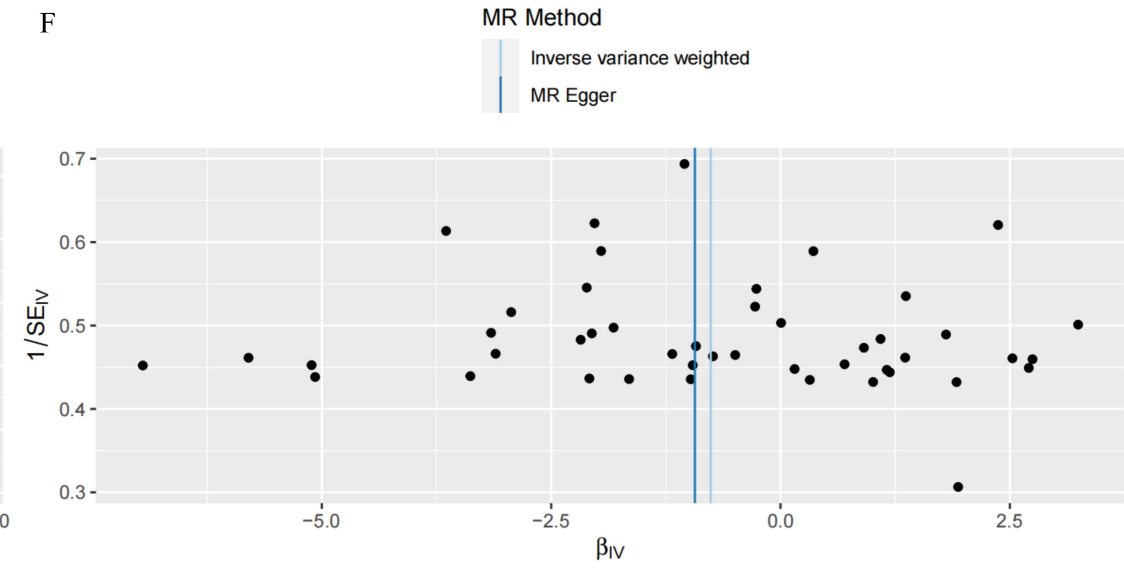

G

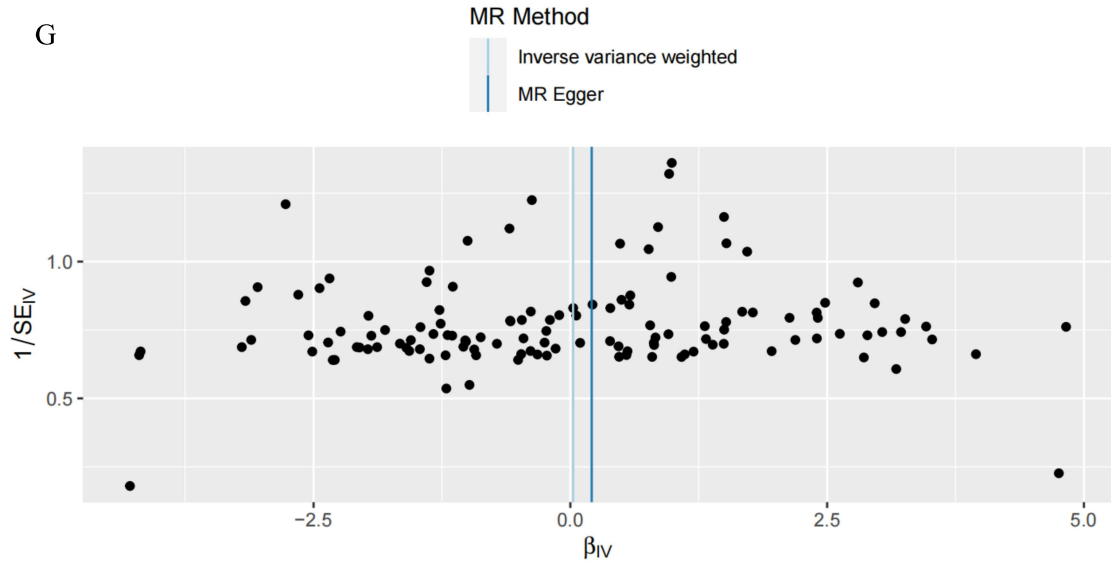

H

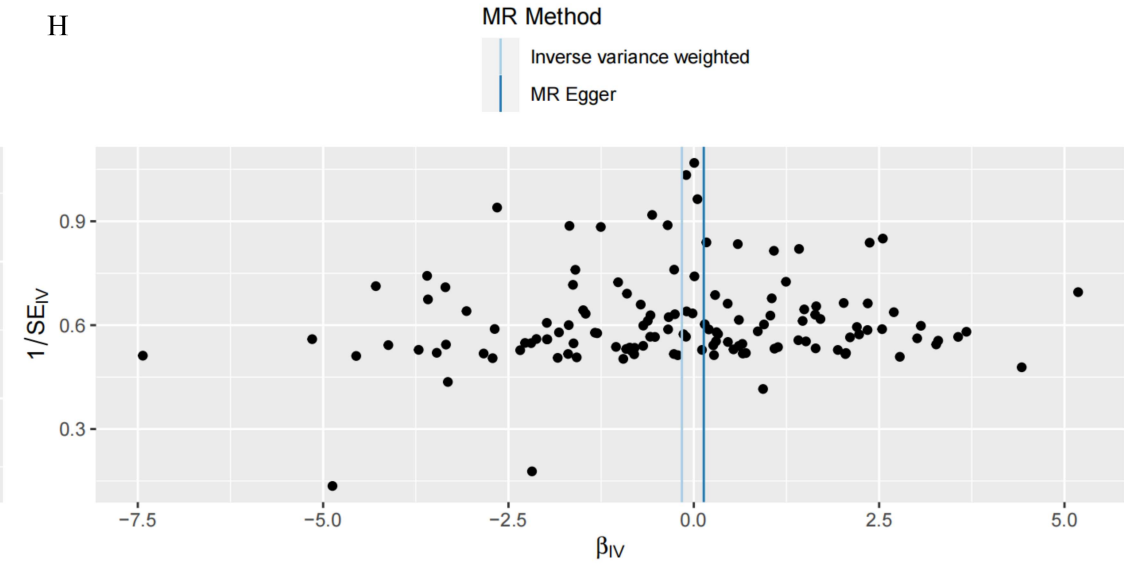

I

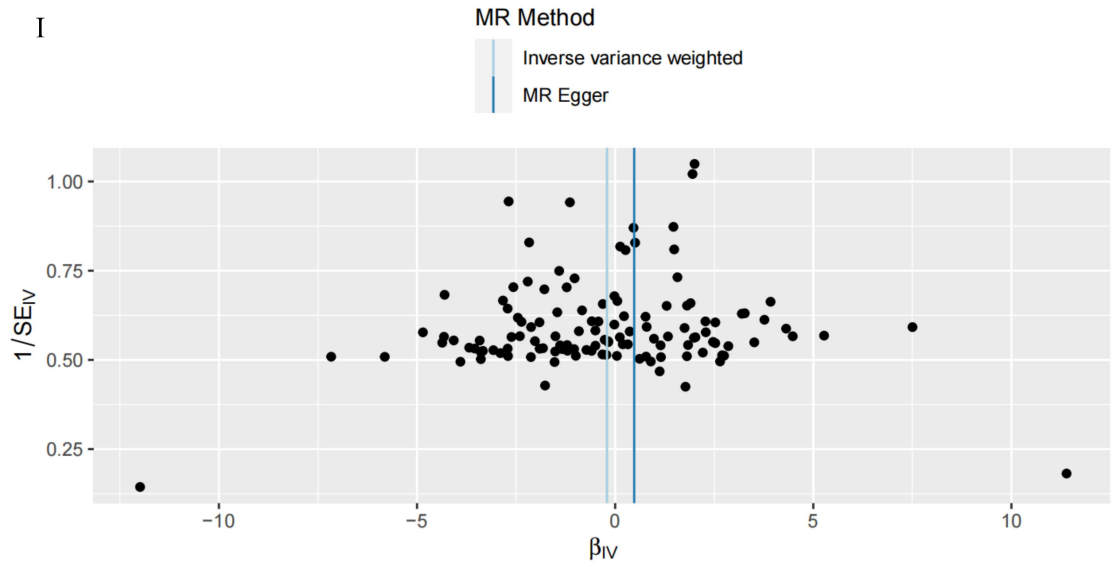

J

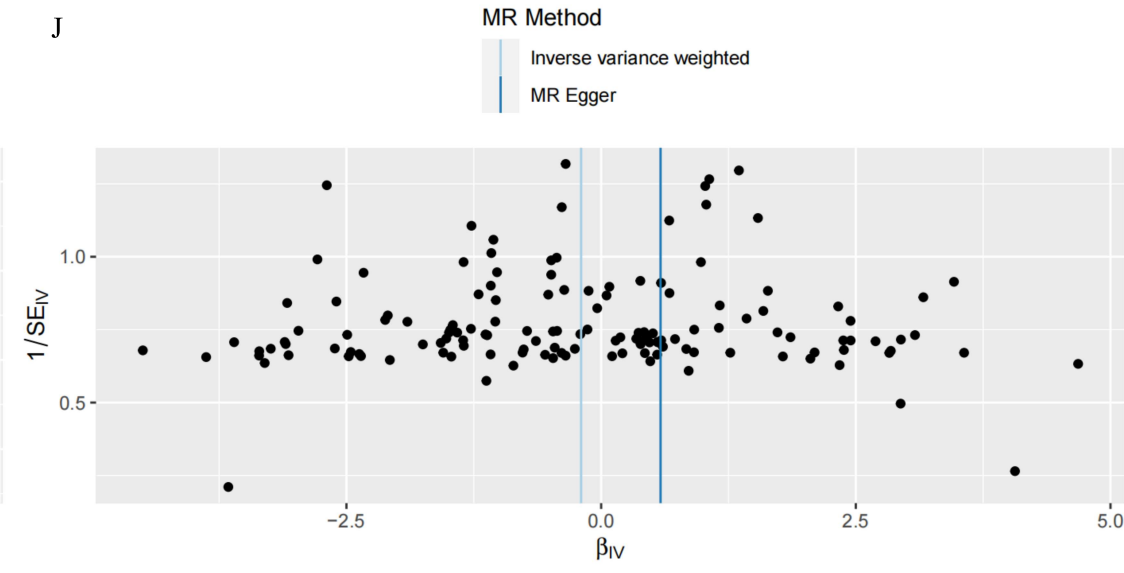

K

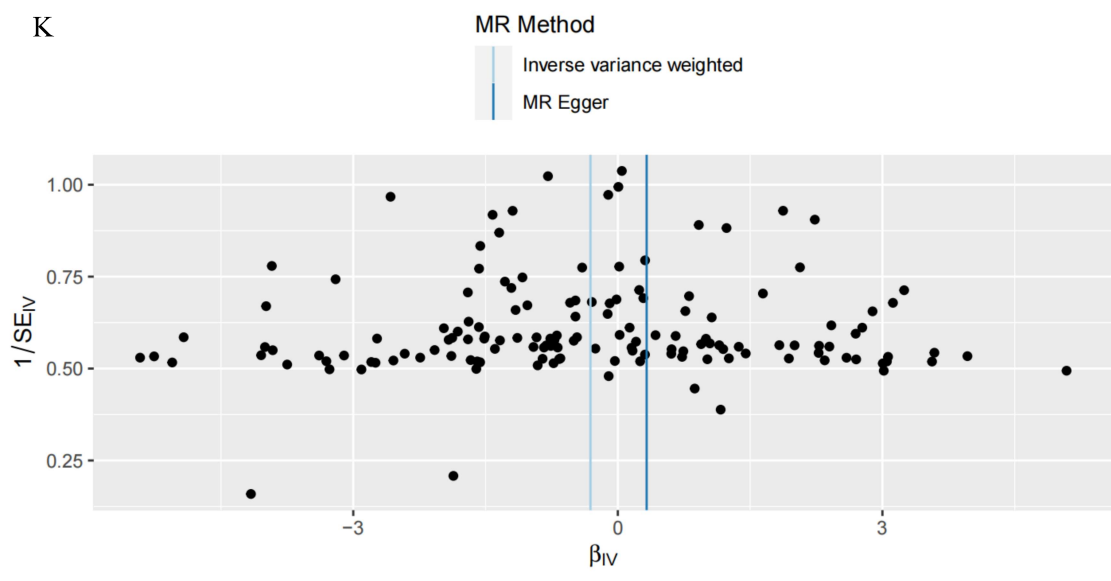

L

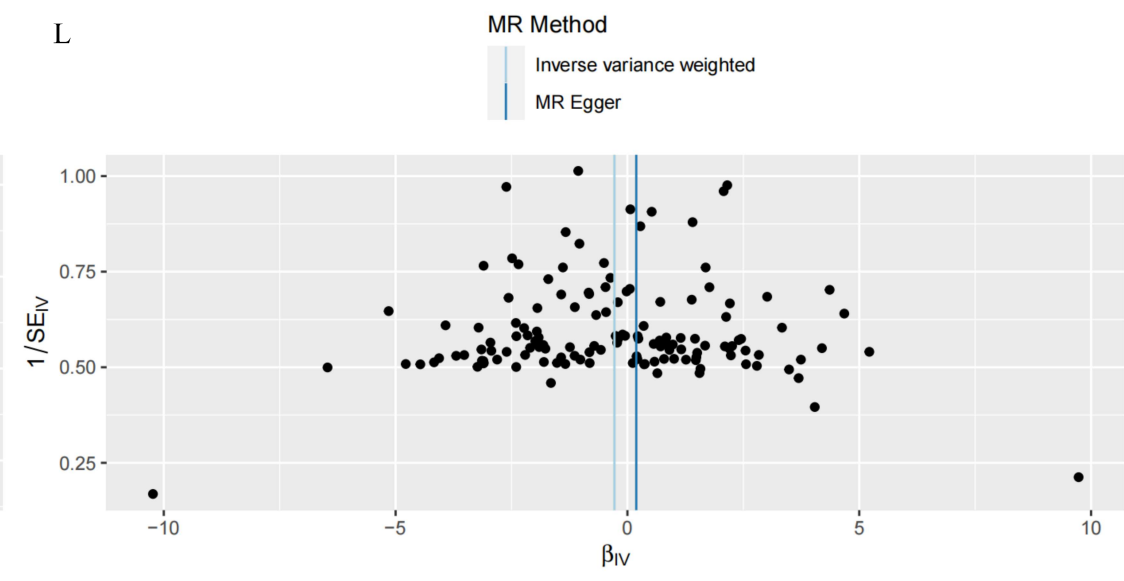

M

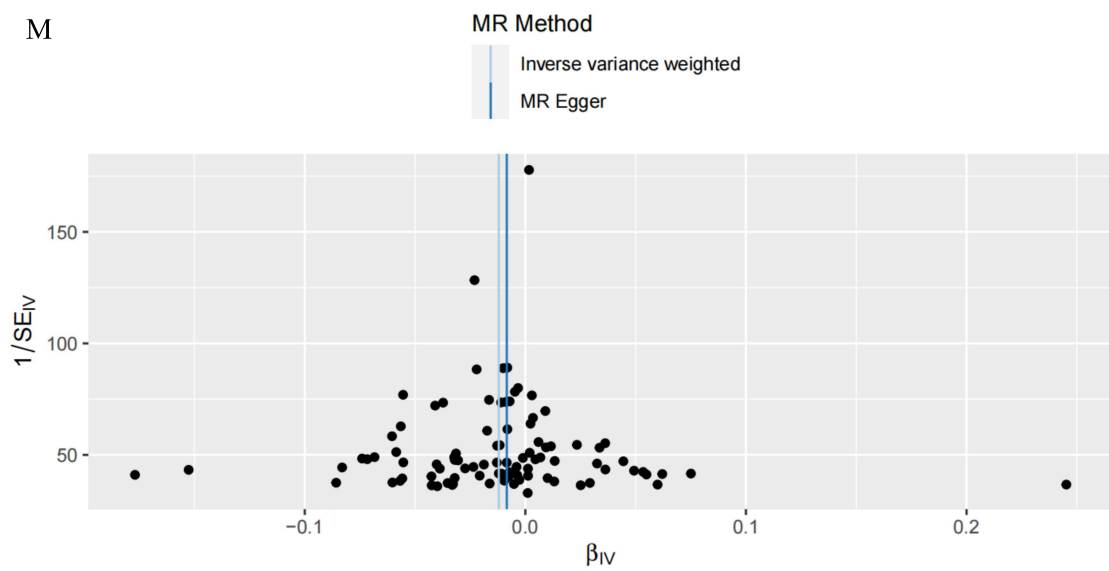

N

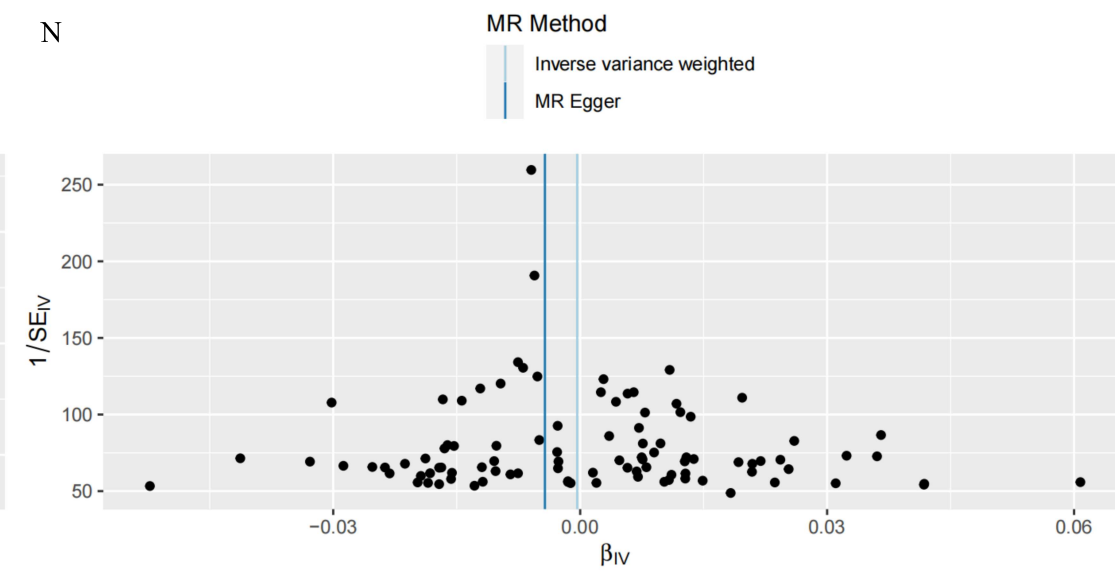

O

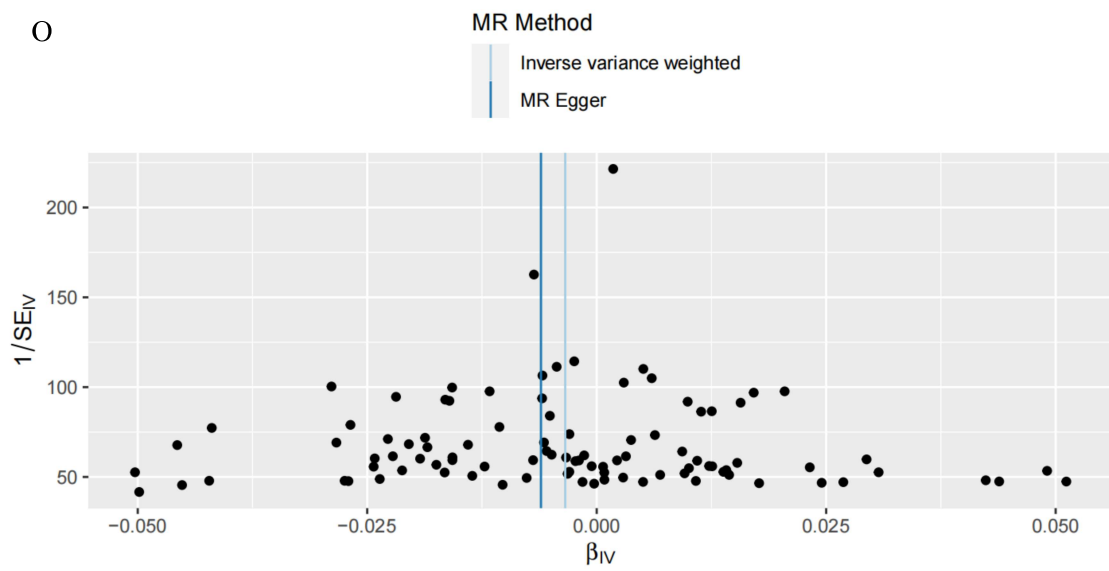

P

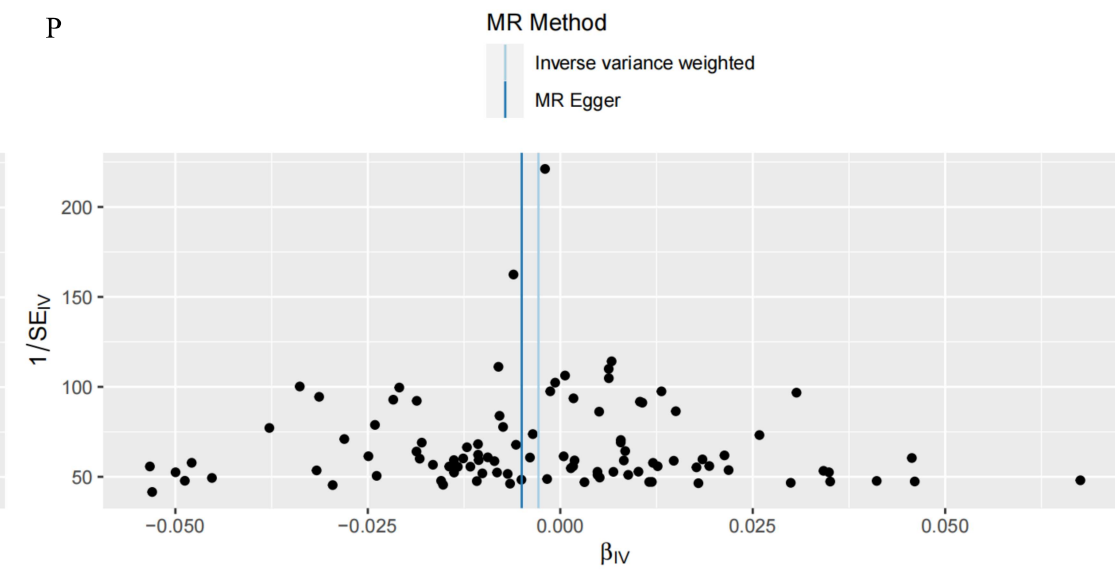

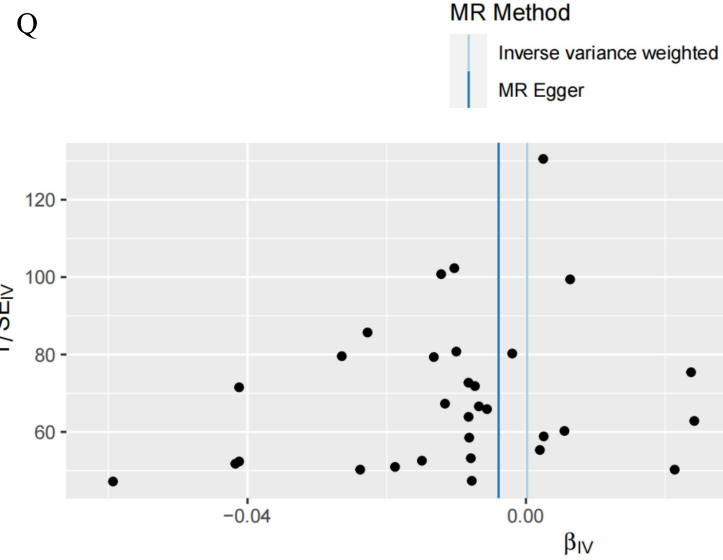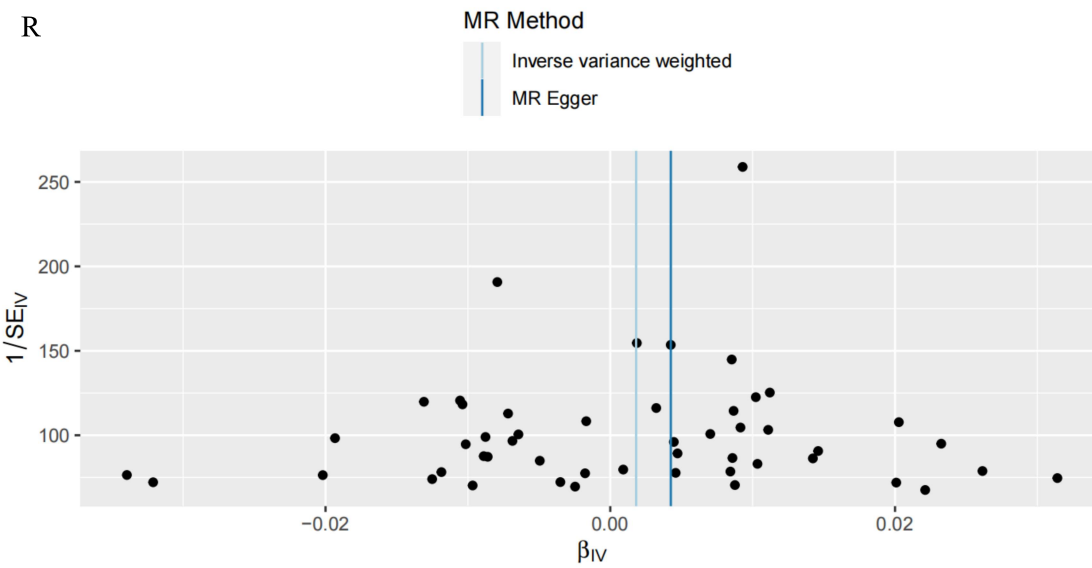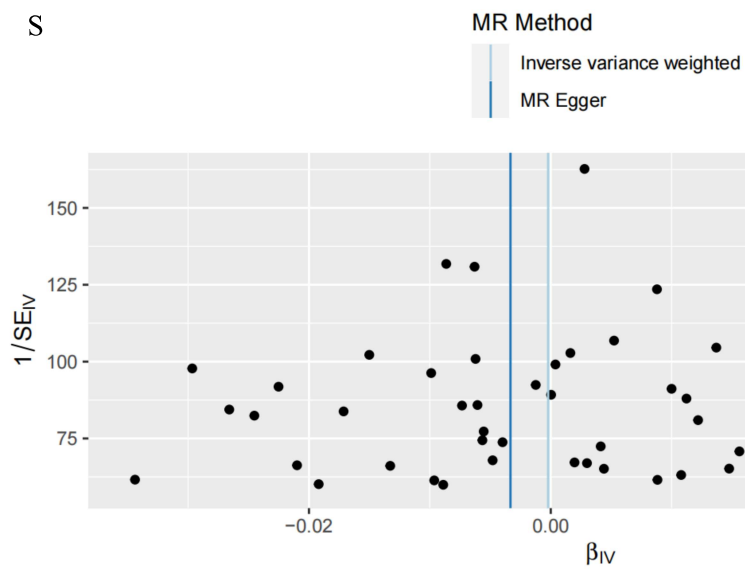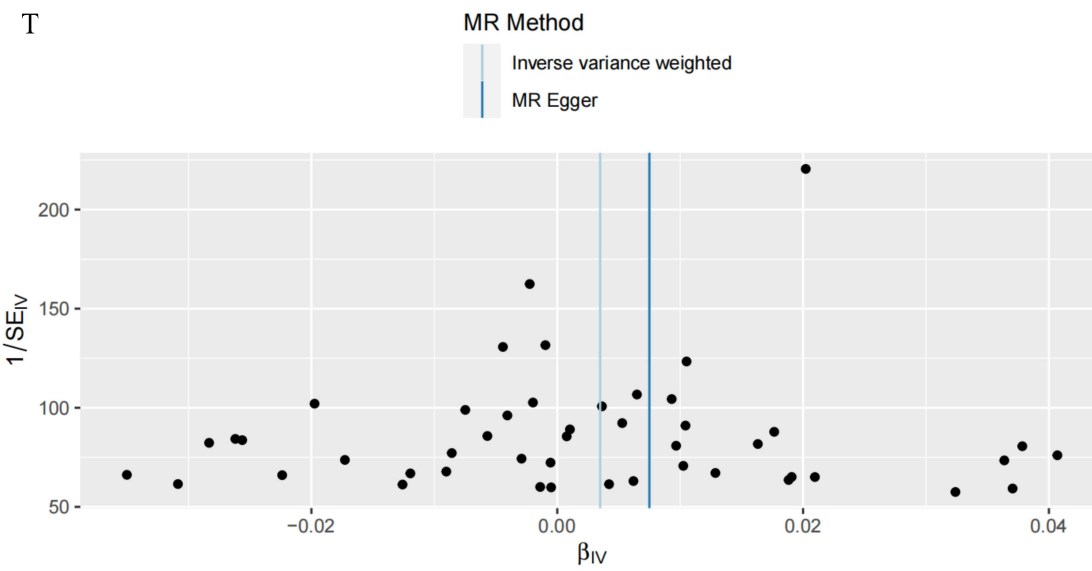

U

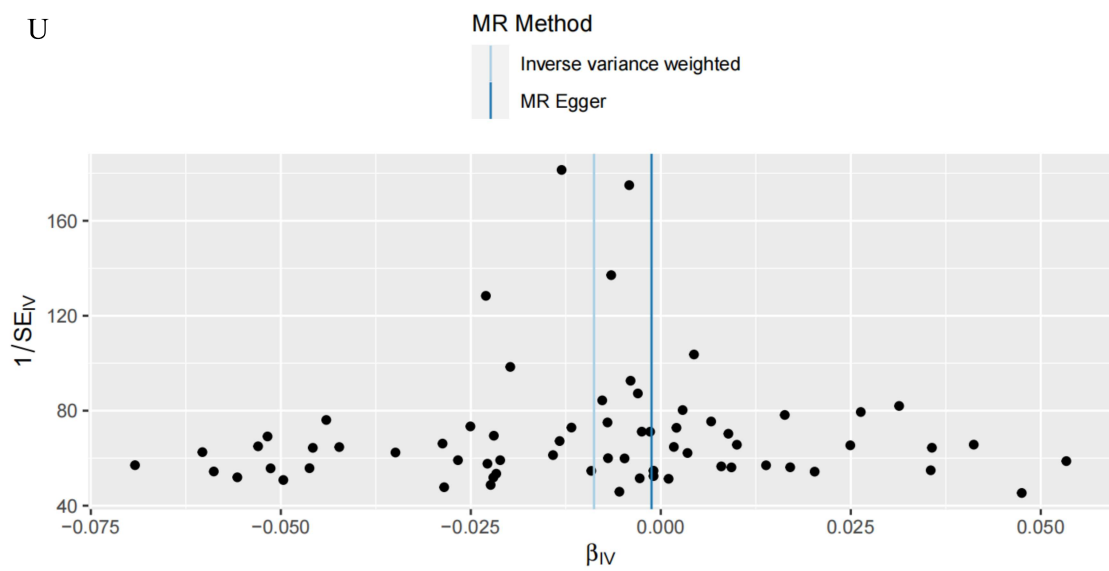

V

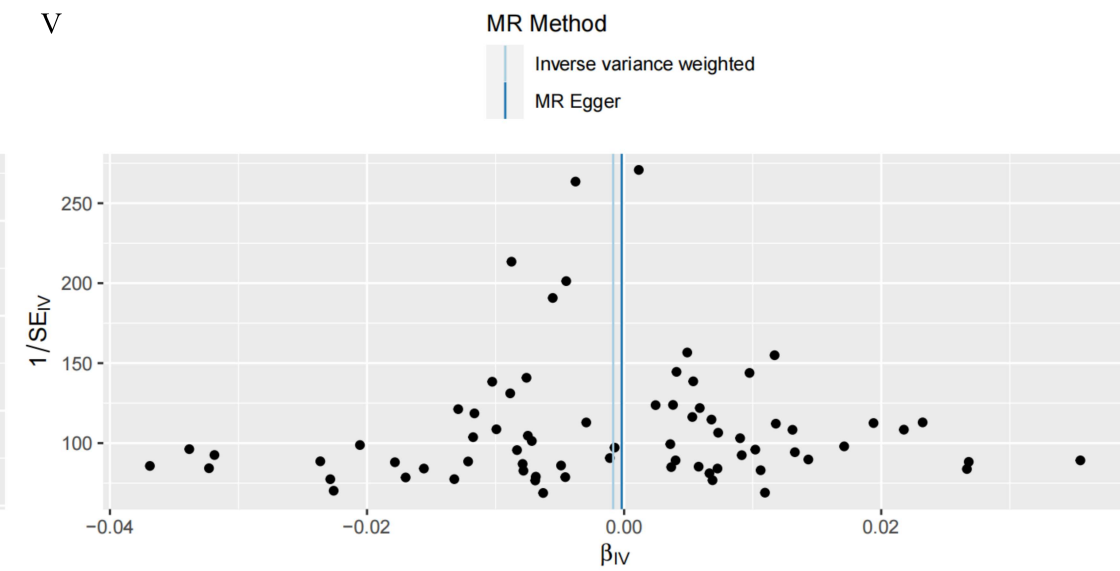

W

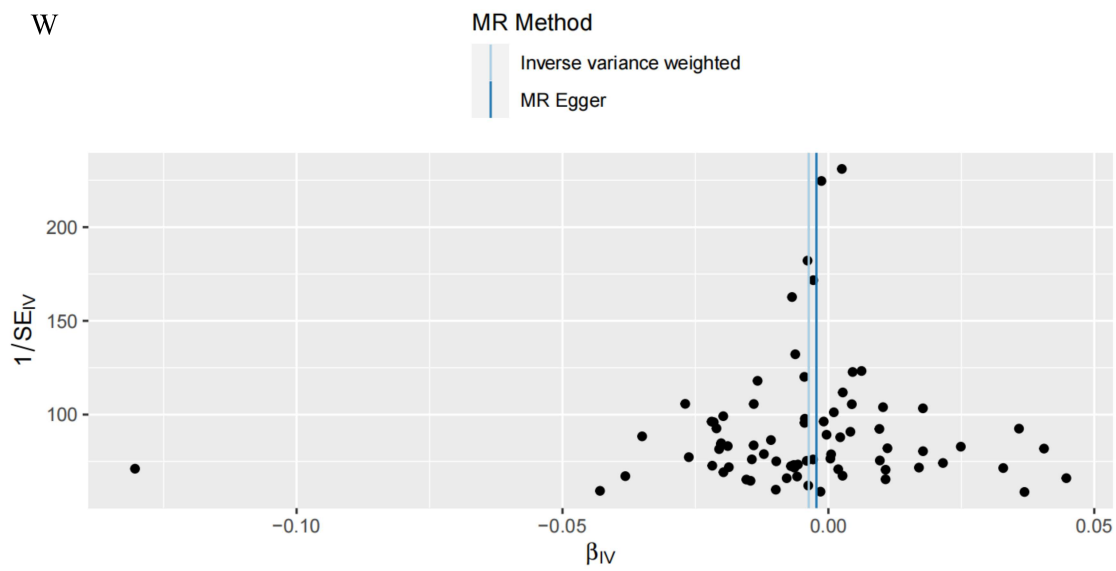

X

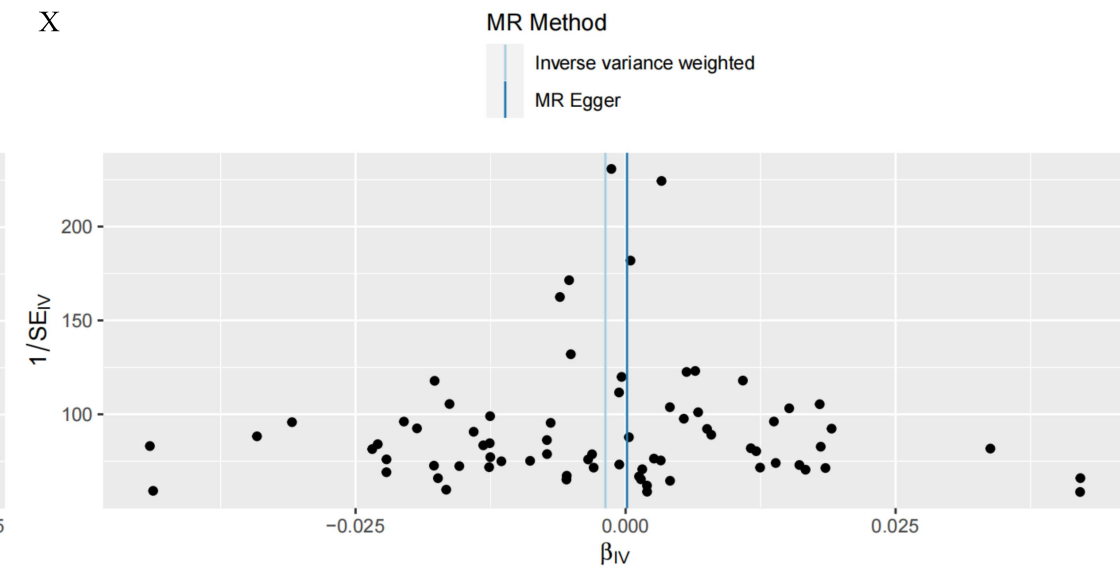

Supplement: Supplementary File 1 — (A–L) Funnel plots of forward MR analysis. (M–X) Funnel plots of reverse MR analysis. [file DataSheet_1.zip › SupMaterial/File 1.pdf]

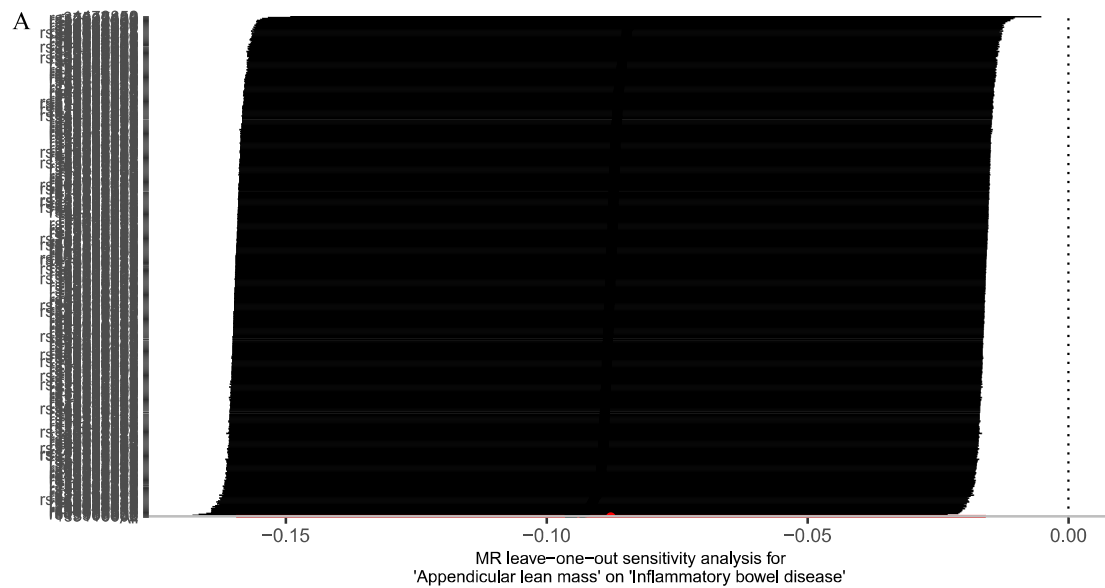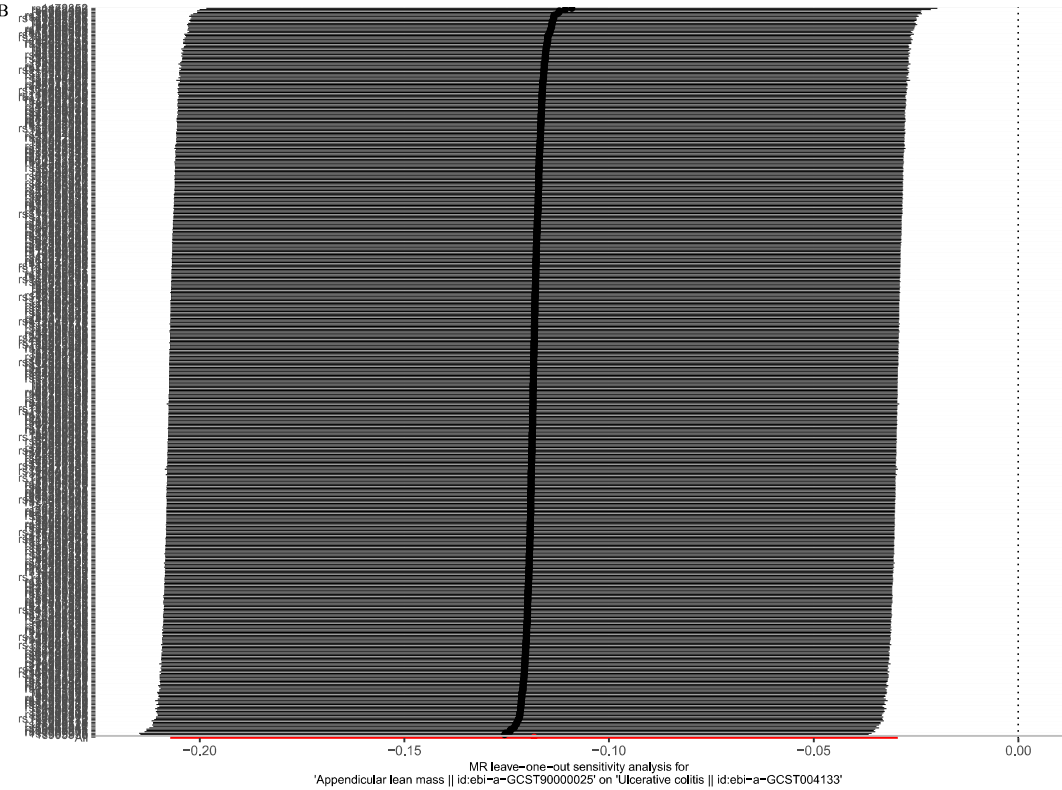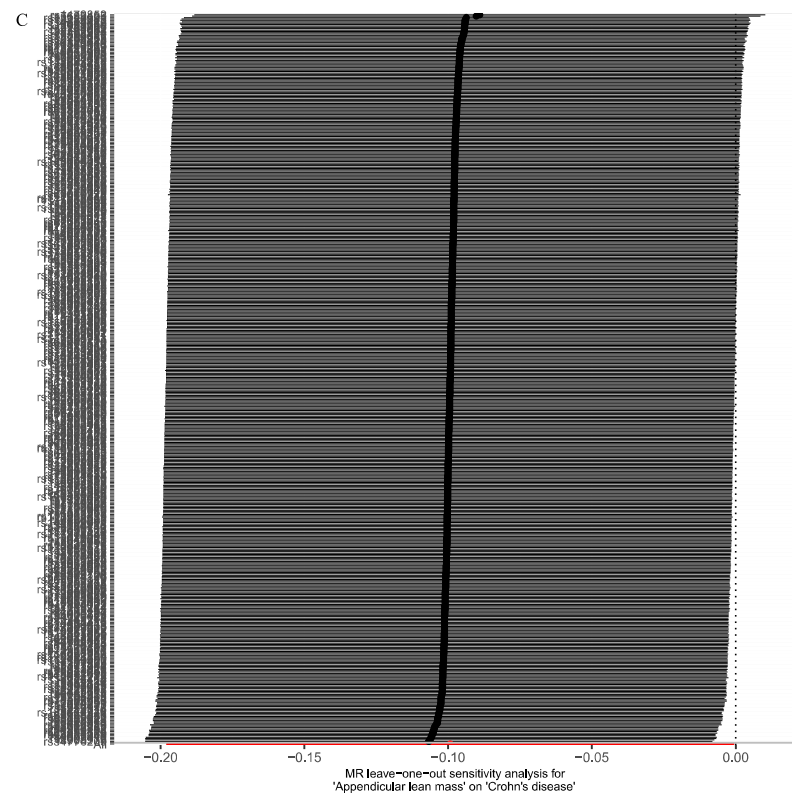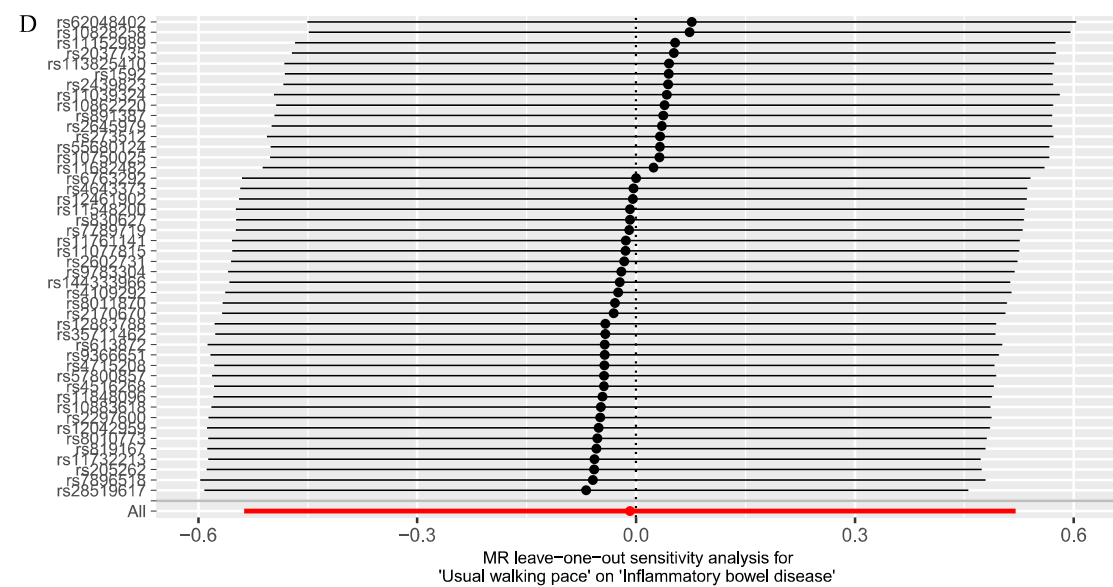

E

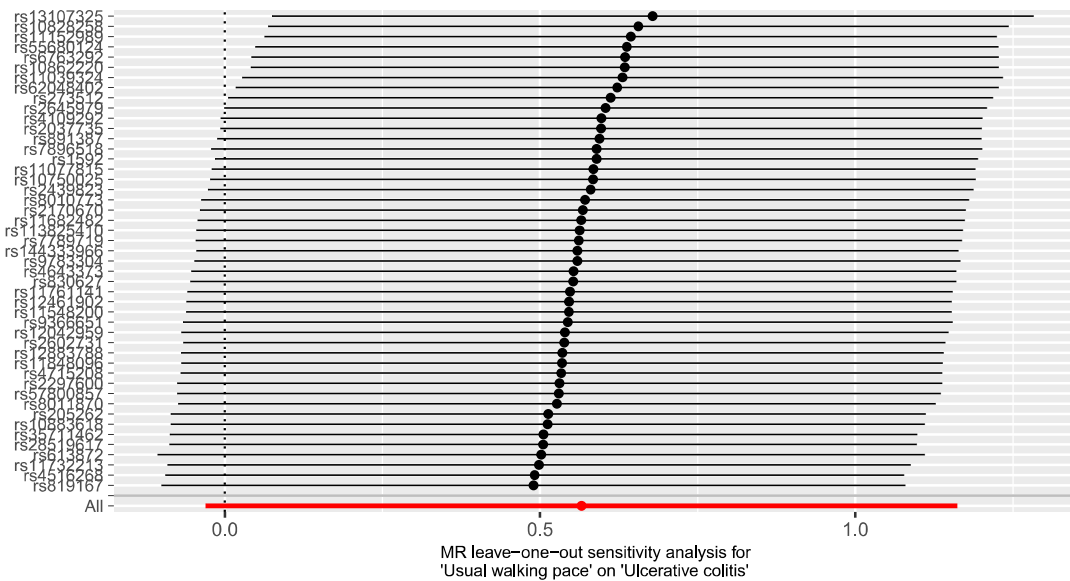

F

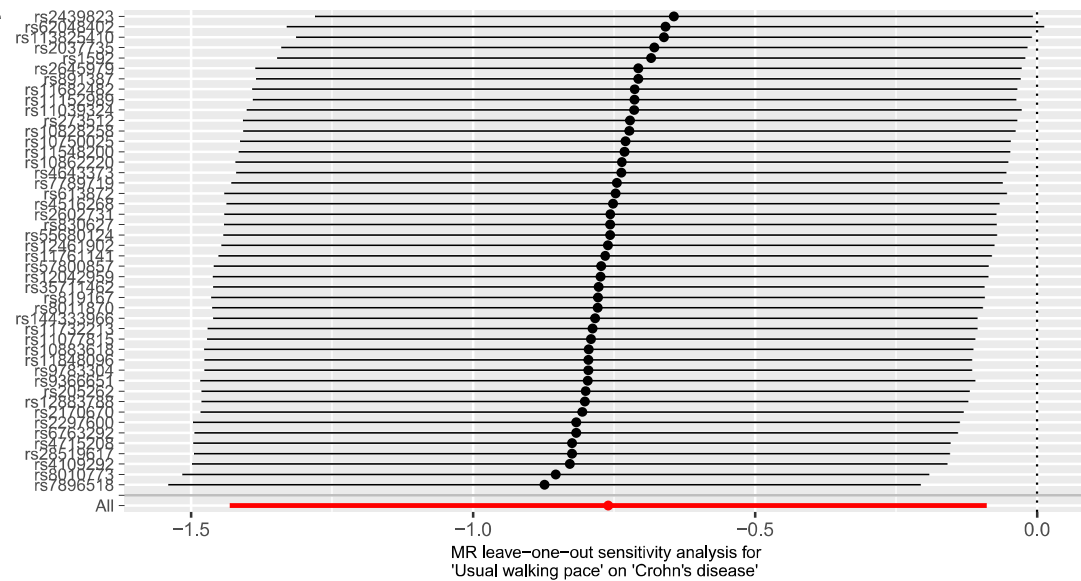

G

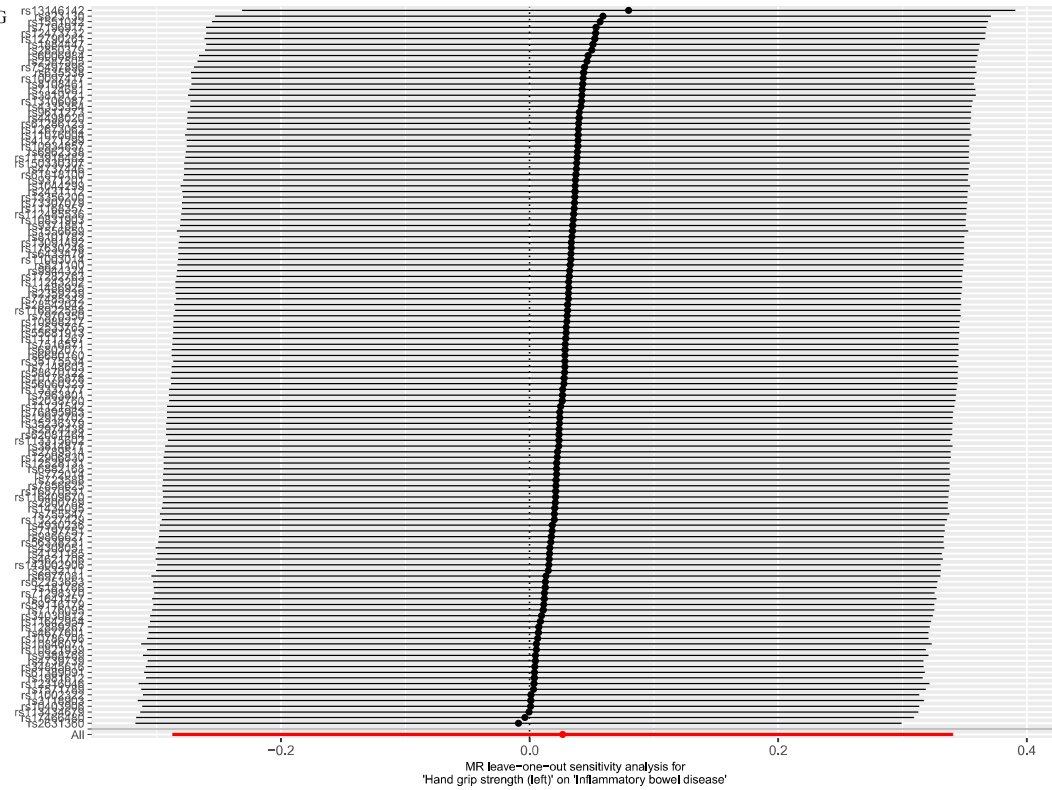

H

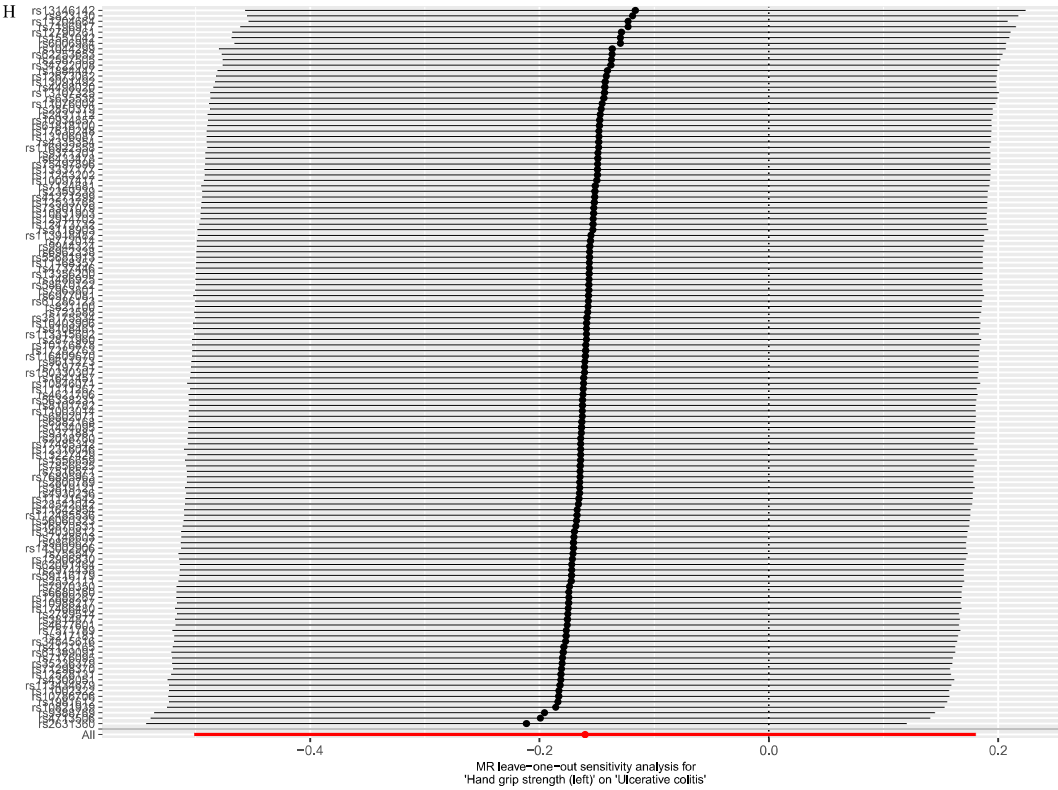

I

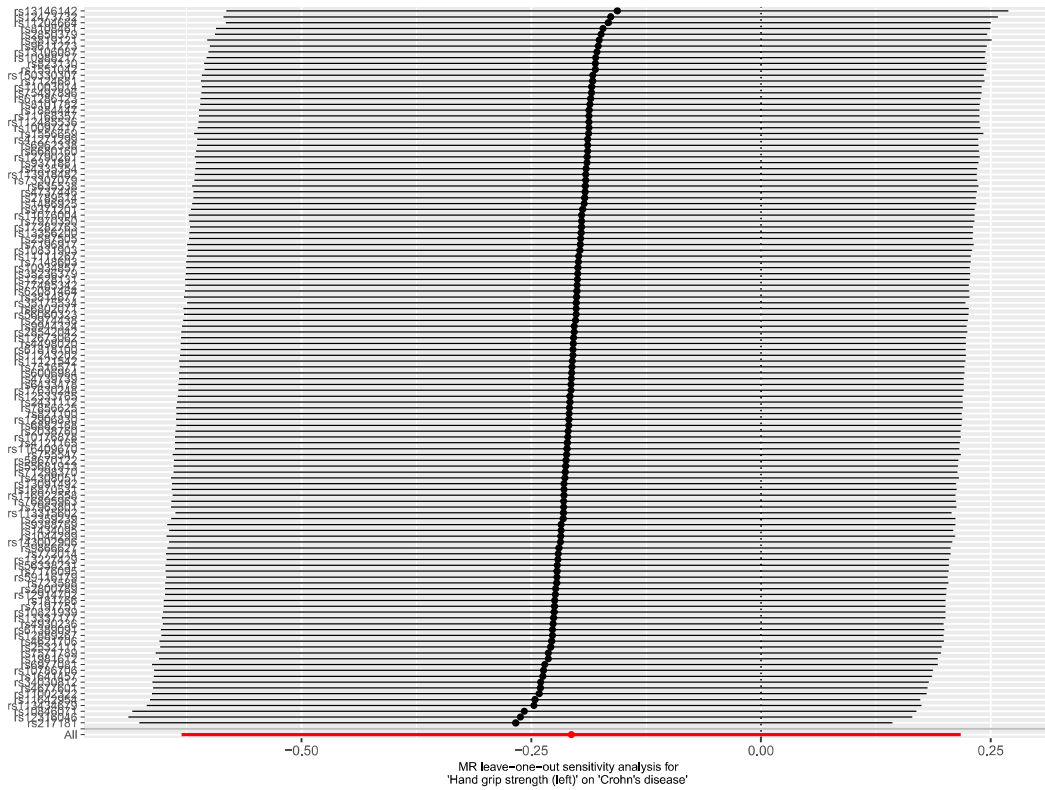

J

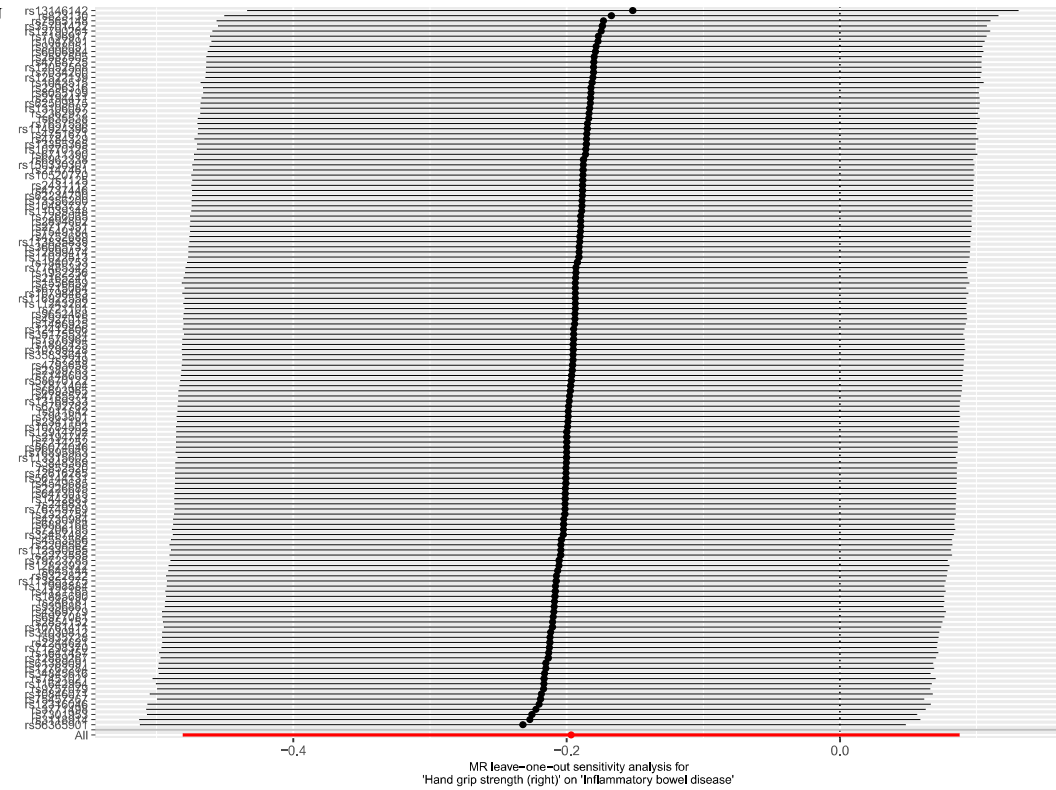

K

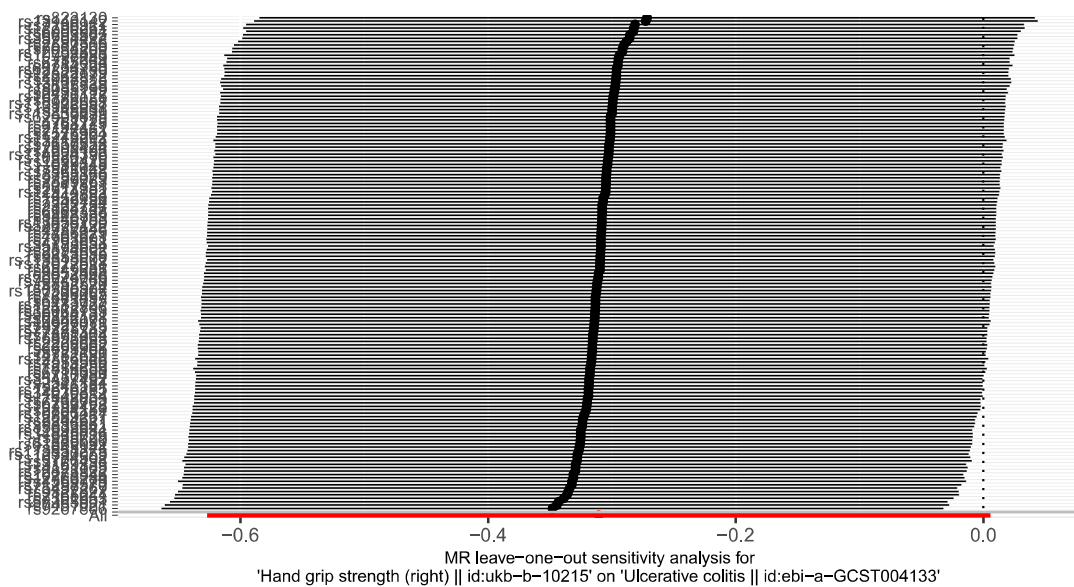

L

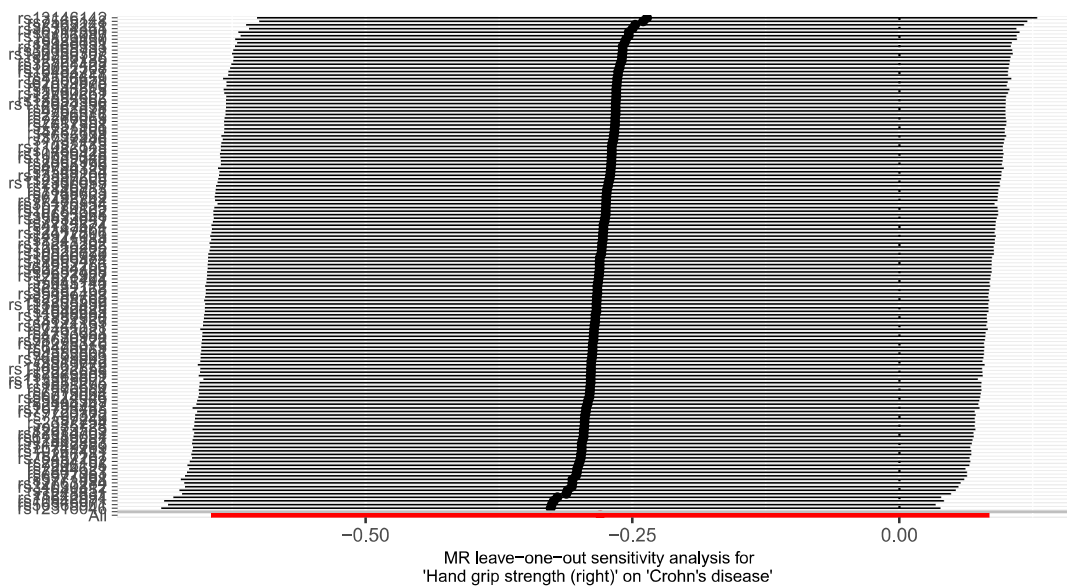

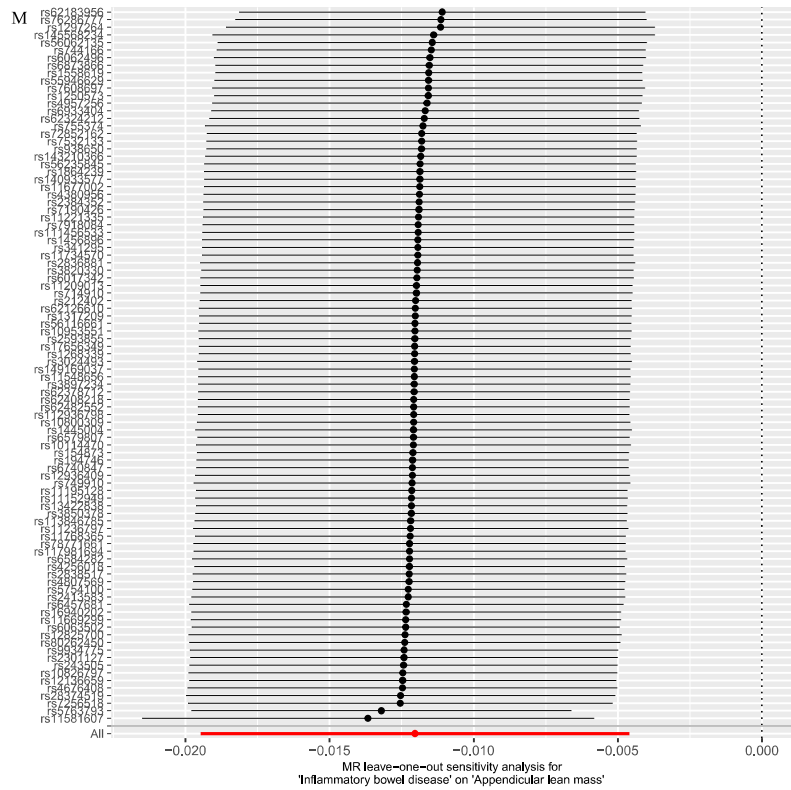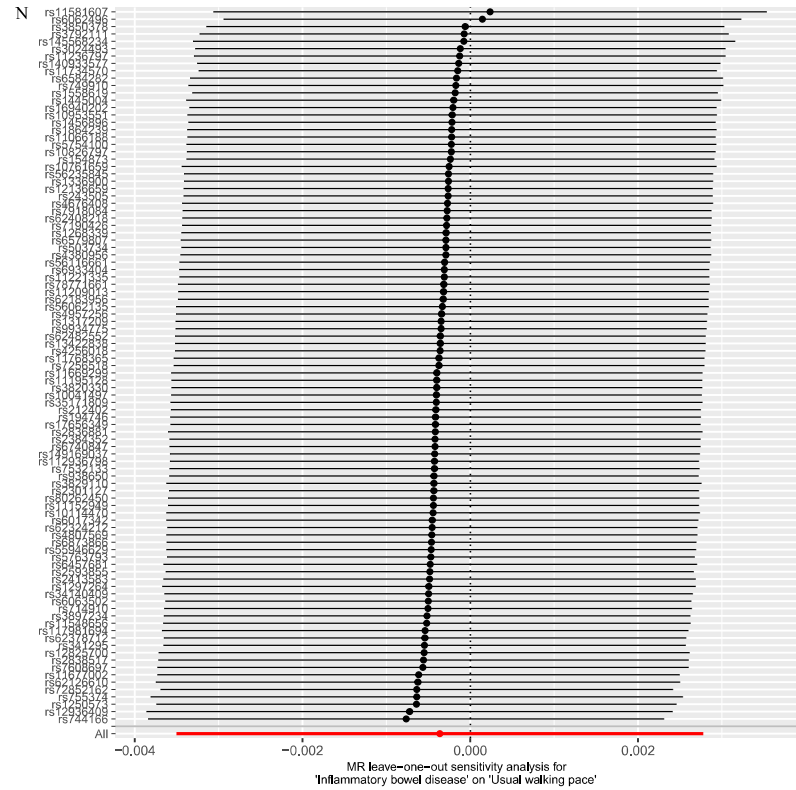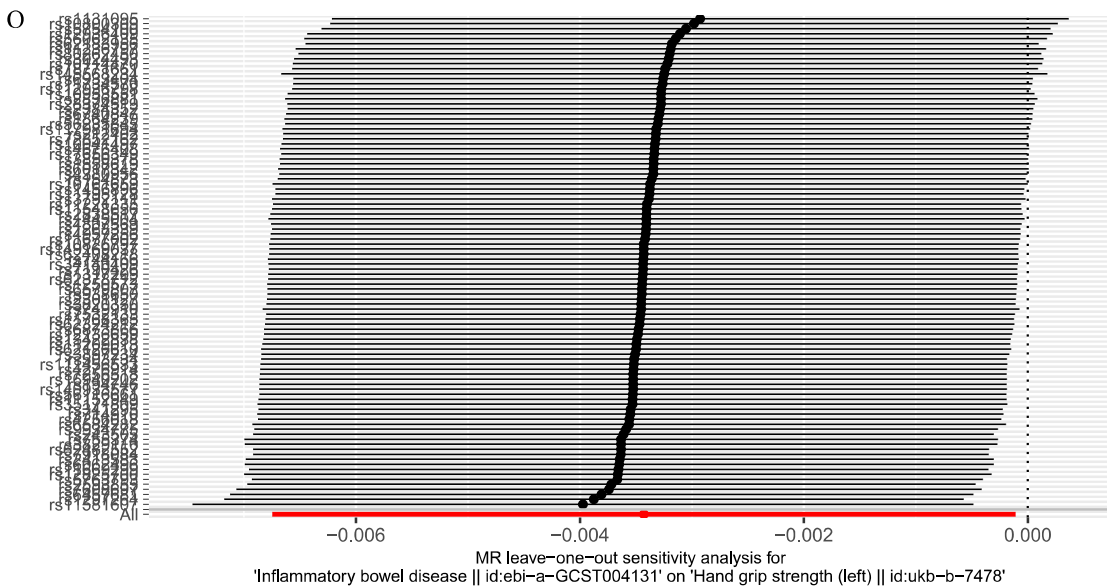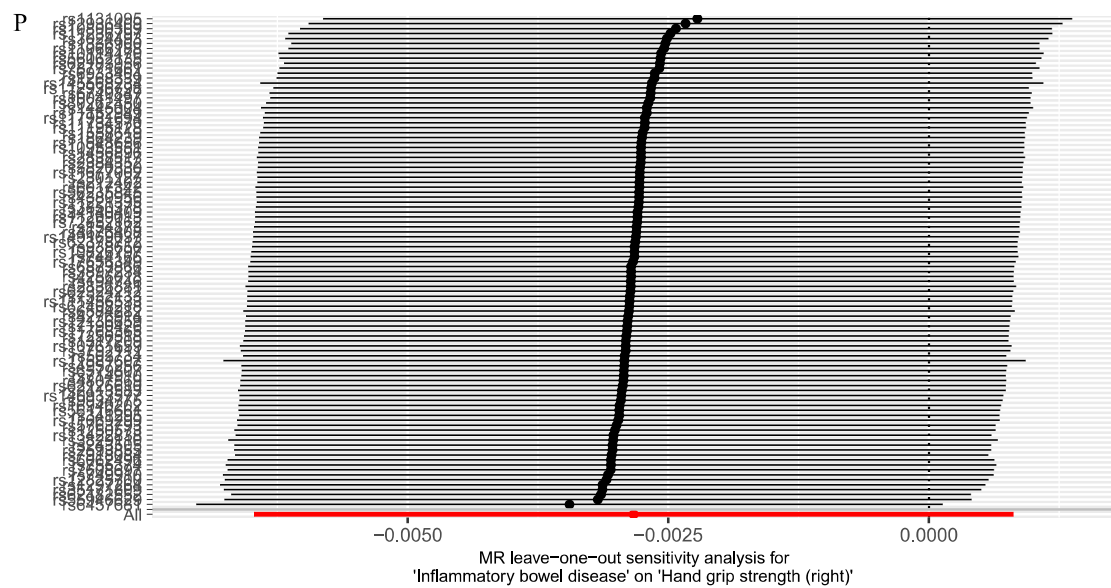

Q

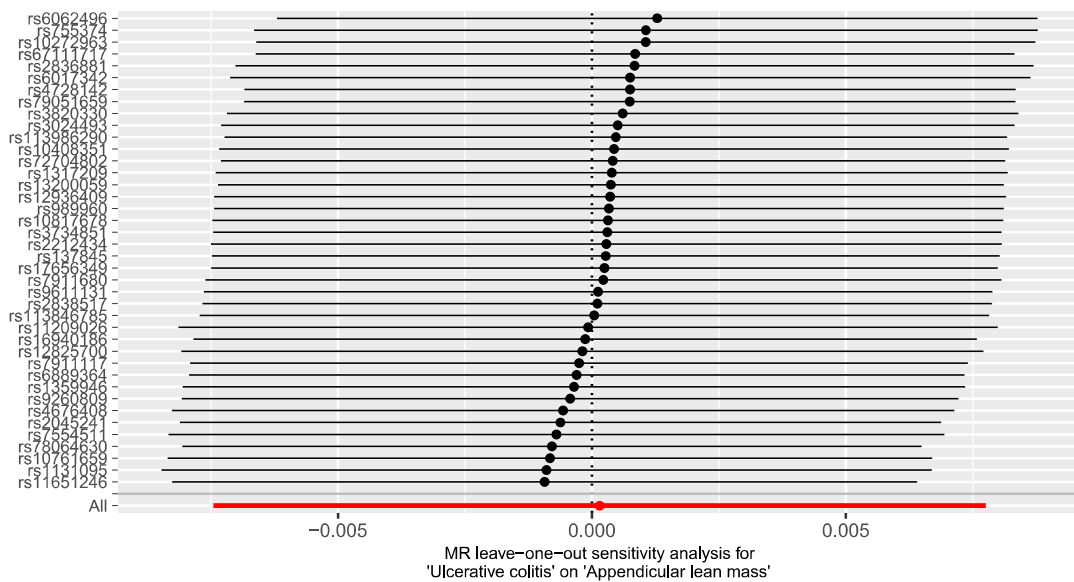

R

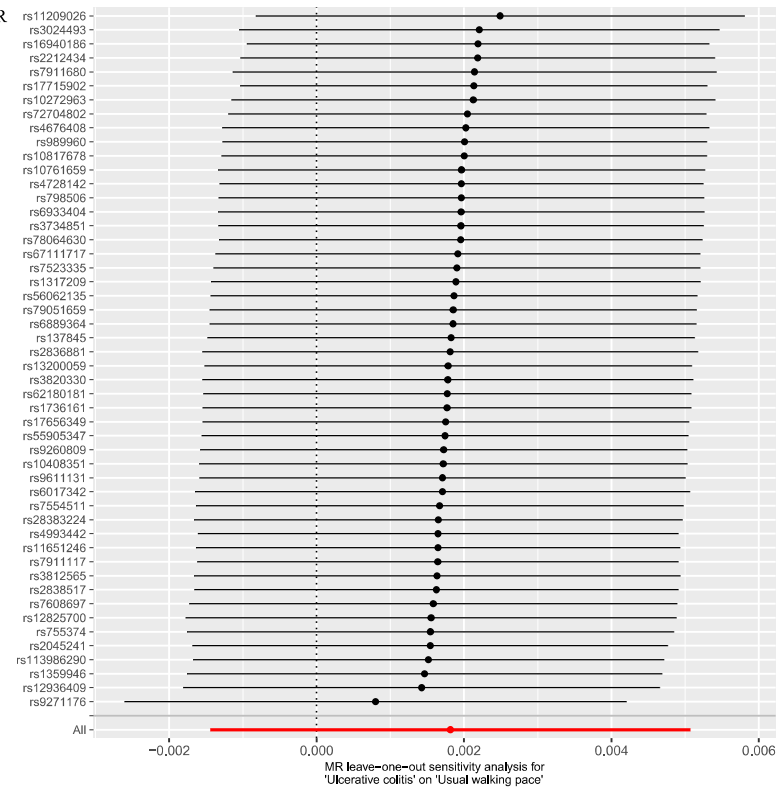

S

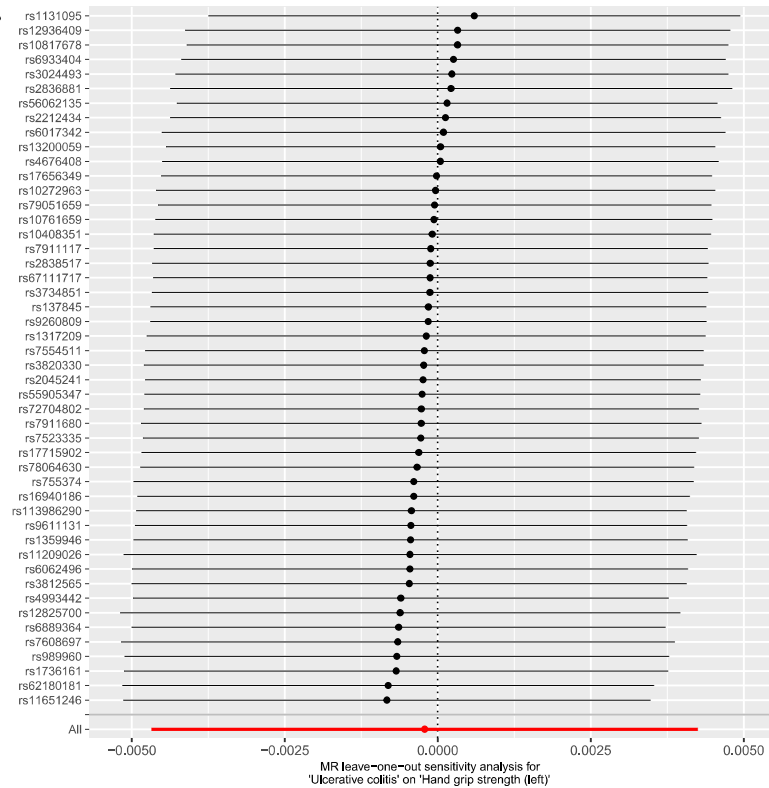

T

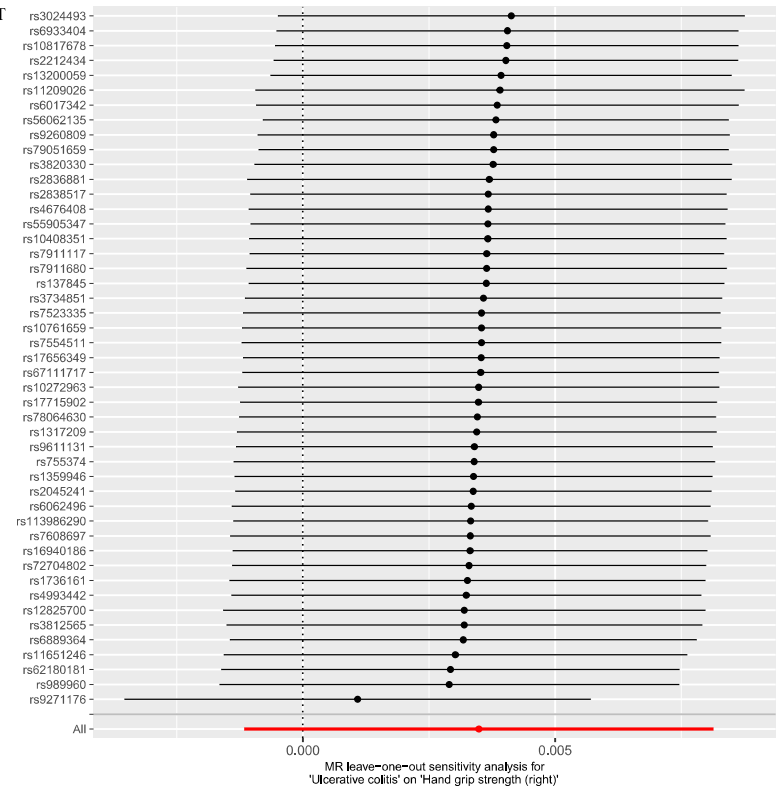

U

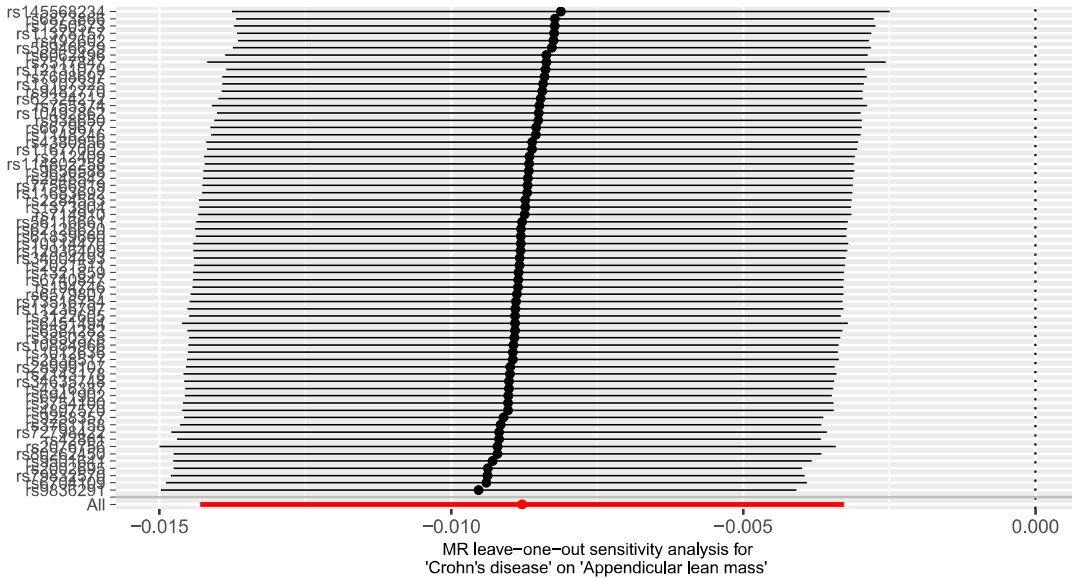

V

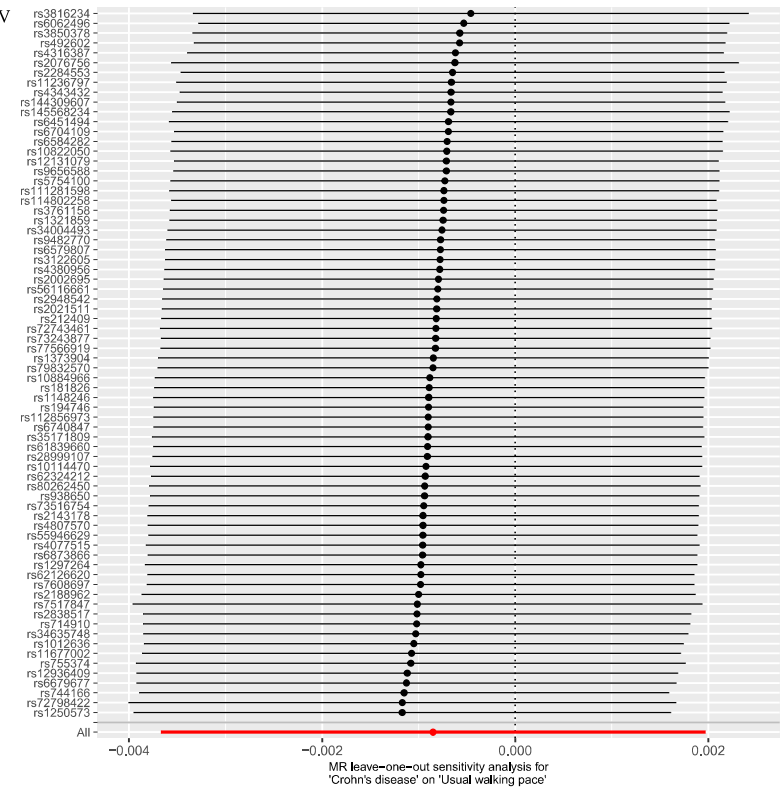

W

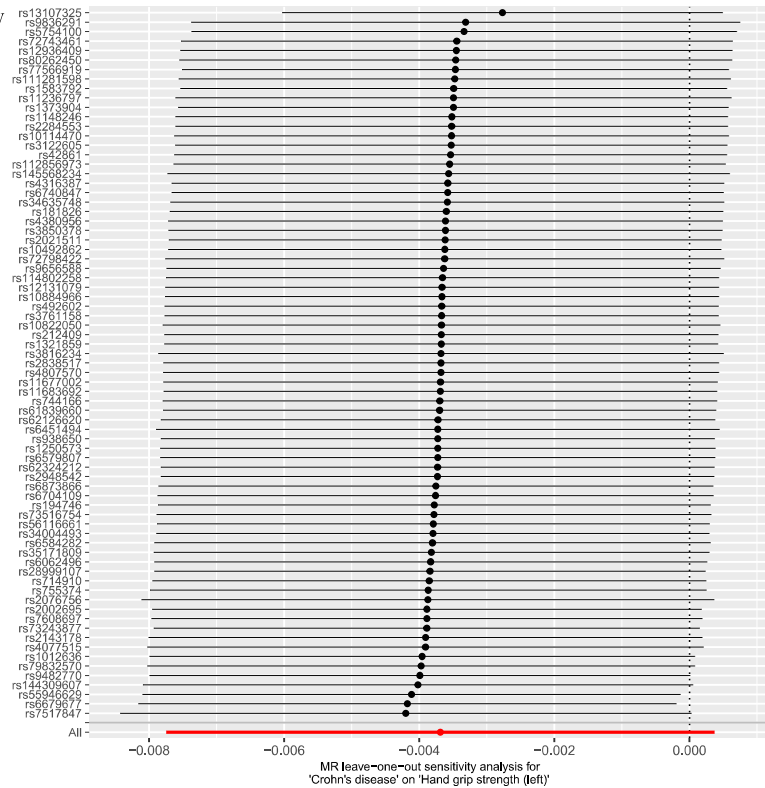

X

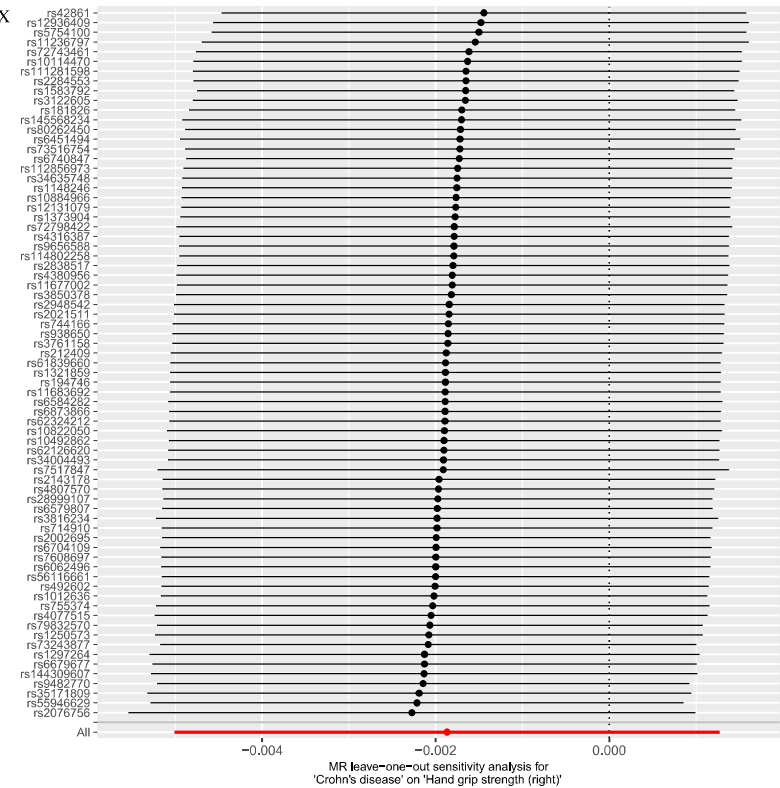

Supplement: Supplementary File 1 — (A–L) Funnel plots of forward MR analysis. (M–X) Funnel plots of reverse MR analysis. [file DataSheet_1.zip › SupMaterial/File 2.pdf]
